# Posttranscriptional Gene Regulatory Networks in Chronic Airway Inflammatory Diseases: *In silico* Mapping of RNA-Binding Protein Expression in Airway Epithelium

**DOI:** 10.3389/fimmu.2020.579889

**Published:** 2020-10-16

**Authors:** Luca Ricciardi, Giorgio Giurato, Domenico Memoli, Mariagrazia Pietrafesa, Jessica Dal Col, Ilaria Salvato, Annunziata Nigro, Alessandro Vatrella, Gaetano Caramori, Vincenzo Casolaro, Cristiana Stellato

**Affiliations:** ^1^Department of Medicine, Surgery and Dentistry Scuola Medica Salernitana, University of Salerno, Salerno, Italy; ^2^Pulmonology, Department of Biomedical Sciences, Dentistry and Morphological and Functional Imaging (BIOMORF), University of Messina, Messina, Italy; ^3^Department of Medicine, Johns Hopkins University School of Medicine, Baltimore, MD, United States

**Keywords:** airway epithelium, chronic inflammation, COPD, posttranscriptional gene regulation, RNA binding proteins, severe asthma

## Abstract

**Background:** Posttranscriptional gene regulation (PTGR) contributes to inflammation through alterations in messenger RNA (mRNA) turnover and translation rates. RNA-binding proteins (RBPs) coordinate these processes but their role in lung inflammatory diseases is ill-defined. We evaluated the expression of a curated list of mRNA-binding RBPs (mRBPs) in selected Gene Expression Omnibus (GEO) transcriptomic databases of airway epithelium isolated from chronic obstructive pulmonary disease (COPD), severe asthma (SA) and matched control subjects, hypothesizing that global changes in mRBPs expression could be used to infer their pathogenetic roles and identify novel disease-related regulatory networks.

**Methods:** A published list of 692 mRBPs [Nat Rev Genet 2014] was searched in GEO datasets originated from bronchial brushings of stable COPD patients (C), smokers (S), non-smokers (NS) controls with normal lung function (*n* = 6/12/12) (GEO ID: GSE5058) and of (SA) and healthy control (HC) (*n* = 6/12) (GSE63142). Fluorescence intensity data were extracted and normalized on the medians for fold change (FC) comparisons. FCs were set at ≥ |1.5| with a false discovery rate (FDR) of ≤ 0.05. Pearson correlation maps and heatmaps were generated using tMEV tools v4_9_0.45. DNA sequence motifs were searched using PScan-ChIP. Gene Ontology (GO) was performed with Ingenuity Pathway Analysis (IPA) tool.

**Results:** Significant mRBP expression changes were detected for S/NS, COPD/NS and COPD/S (*n* = 41, 391, 382, respectively). Of those, 32% of genes changed by FC ≥ |1.5| in S/NS but more than 60% in COPD/NS and COPD/S (*n* = 13, 267, 257, respectively). Genes were predominantly downregulated in COPD/NS (*n* = 194, 73%) and COPD/S (*n* = 202, 79%), less so in S/NS (*n* = 4, 31%). Unsupervised cluster analysis identified in 4 out of 12 S the same mRBP pattern seen in C, postulating subclinical COPD. Significant DNA motifs enrichment for transcriptional regulation was found for downregulated RBPs. Correlation analysis identified five clusters of co-expressed mRBPs. GO analysis revealed significant enrichments in canonical pathways both specific and shared among comparisons. Unexpectedly, no significant mRBPs modulation was found in SA compared to controls.

**Conclusions:** Airway epithelial mRBPs profiling reveals a COPD-specific global downregulation of RBPs shared by a subset of control smokers, the potential of functional cooperation by coexpressed RBPs and significant impact on relevant pathogenetic pathways in COPD. Elucidation of PTGR in COPD could identify disease biomarkers or pathways for therapeutic targeting.

## Introduction

RNA-binding proteins (RBPs) are key regulatory factors in post-transcriptional gene regulation (PTGR) involved in the maturation, stability, transport and degradation of cellular RNAs. RBP convey PTGR by binding to conserved regulatory elements shared by subsets of transcripts and by directing the bound targets toward cytoplasmic sites of translation or decay (1). Importantly, RBPs exert their function as part of ribonucleoprotein (mRNP) complexes, constituted by proteins and non-coding RNAs, such as microRNAs (2). Through stimulus-dependent cues, changes in mRBP composition ultimately determine the rate of target mRNA stability and translation. Therefore, understanding RBP function in disease models requires a larger evaluation of co-expression and regulatory scenarios, shaped by disease-driven triggers and signaling.

For human cancer the occurrence of aberrant RBP expression, along with altered RNA turnover and translation rates have been characterized in preclinical models, identified in human disease and further probed with *in silico* approaches (3, 4) leading to the identification of this class of regulators as novel disease biomarkers and as targets for small molecule-based therapeutics (5–7). Similar studies in human chronic inflammatory diseases are lagging behind, despite ample knowledge of deregulated PTGR in inflammatory responses by *in vitro* studies and the strong inflammatory phenotypes obtained in some of the knock-out animal models (8, 9). This knowledge chasm is also present for lung diseases, despite the strong link between chronic inflammatory diseases such as Chronic Obstructive Pulmonary Disease (COPD) and some lung cancer types (10) and the extensive overlap of RBP-regulated genes contributing to both disease process (11).

Airway epithelium is a major driver in the pathogenesis of inflammatory lung diseases such as asthma and COPD. Deregulated epithelial responses are a main therapeutic target of inhaled corticosteroids (ICS), the mainstay anti-inflammatory drug class for asthma symptom control (www.ginasthma.org) and for treating exacerbations in COPD (www.goldcopd.org). Involvement of RBPs in airway epithelial responses to inflammatory stimuli and glucocorticoid treatment has been well-characterized *in vitro* (12–15) but awaits evidence from patient-based studies. To this end, in a previous characterization of ARE-binding proteins expression in COPD (16) we identified a selective loss of the RBP AU-binding Factor (AUF)−1 in bronchial biopsies in stable COPD patients vs. smoker controls. Besides replicating this finding *in vitro* upon challenge of the epithelial cell line BEAS-2B with inflammatory cytokines and cigarette smoke extract, we confirmed this loss in primary epithelium, by interrogating a transcriptome database generated from bronchial brushings of COPD patients vs. normal smokers and non-smokers controls (17).

On these basis, we hypothesized that changes in mRNA-binding RBPs (mRBP) expression may occur on a larger scale in chronic inflammatory airways diseases, such as COPD and severe asthma (SA) and that identification of these changes can be used to infer their putative pathogenetic roles as disease-related regulatory networks, as in cancer (18). We therefore set out to evaluate the expression profile of a curated gene list of mRBPs in selected public Gene Expression Omnibus (GEO) transcriptomic databases derived from airway epithelium isolated from bronchial brushings of phenotyped patients affected by COPD, SA and relative control populations, enrolled in relevant translational studies on disease pathophysiology. In particular, we employed for COPD the same database (17) in which we found loss of AUF-1 in COPD samples vs. controls, as observed *ex vivo* in COPD airway biopsies (16) and a GEO database from SA and healthy controls (HC) enrolled in the Severe Asthma Research Program (SARP) study (19). The list of mRBPs we utilized for this study has been created by annotation of proteins as RBPs generated by domain search, considering known RNA binding domains [among 800 domains extracted from the protein family (Pfam) database], or including proteins known as validated partners of RNP complex (20). The mRBP expression profiles we obtained were then evaluated through gene ontology analysis to evaluate their potential relevance to disease pathogenesis; moreover, we searched for coregulated RBP expression indicative of common participation to regulatory pathways.

In airway epithelial samples from COPD patients, this approach identified a global downregulation of RBP expression that was shared by a subset of smoker control subjects; changes in mRBP expression impacted several biological pathways also involved in several aspects of COPD pathogenesis; finally, at least five groups of coregulated RBPs were identified. Importantly, airway epithelial mRBP expression was found to be much less regulated in patients with SA.

## Materials and Methods

### Sample Selection and Data Processing

**Figure 1:**
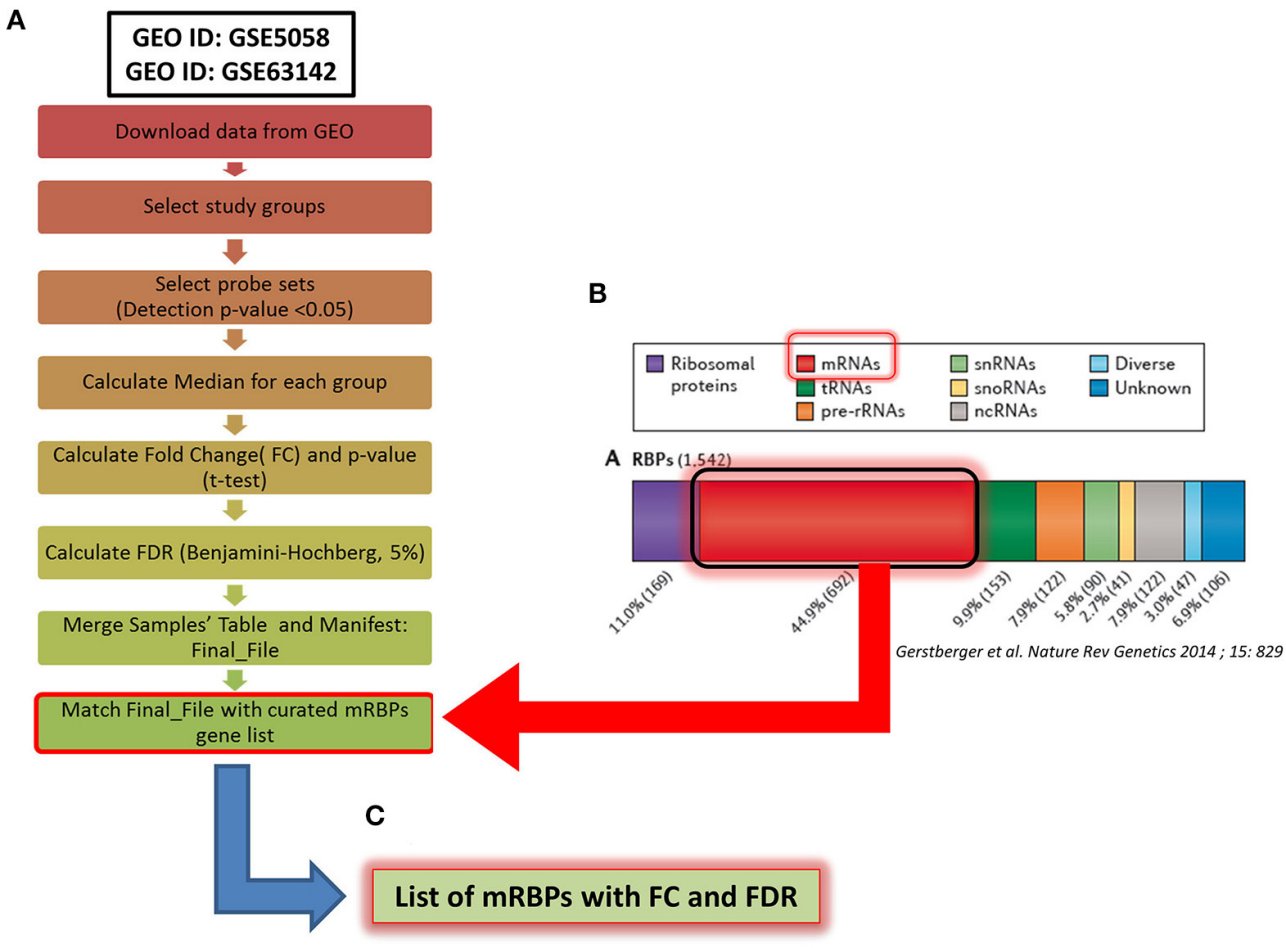
Methodological flowchart of the study for expression profiling of mRNA-binding proteins (mRBPs) in airway epithelium transcriptomic studies in COPD and severe asthma (SA). **(A)** Flowchart for data analysis of the trascriptomic datasets obtained in the Gene Expression Omnibus (GEO) public database (see Methods). Briefly, fluorescence intensity data (raw data) from the chosen datasets (NS, S, stable COPD for GSE5058; SA and HC for GSE63142) were extracted and processed to calculate the probes fluorescence compared to the background. Probes with at least one detection *p* < 0.05 in all three groups were considered for further analysis. Data were then normalized by the median to calculate fold change (FC) expression among groups (S/NS, COPD/NS, COPD/S; SA/HC); only those showing a False Discovery Rate (FDR) ≤ 0.05 in each comparison were considered, forming a Final File for each GSE. Concurrently, a curated gene list of 692 RBPs binding to mRNA (mRBPs) was downloaded from a published census of RNA-binding proteins (20) **(B)** and searched in the Final File, producing a dataset of mRBPs with statistically significant fold changes in disease state vs. controls **(C)**.

We selected the following two trascriptomic databases generated from human airway epithelial cells obtained by bronchial brushings, downloaded from the GEO public repository of high-throughput gene expression data (Figure 1A):

- **GEO ID: GSE5058**. COPD patients (C), Smokers (S) and Non-Smokers (NS) as controls with normal al lung function (NLF) (*n* = 6/12/12, respectively) (17);
- **GEO ID: GSE63142**. Patients with severe Asthma (SA), healthy control subjects (HC) (*n* = 6/12, respectively, randomly selected from databes to match number of cases considered in GSE5058) **(****19****)**.
- **GEO ID: GSE8545**. Patients with stable COPD (C), Smokers (S) and Non-Smokers (NS) as controls with normal lung function (NLF) (*n* = 18/18/18, respectively) (21). This dataset has been utilized to validate the RBP expression profile in COPD/S identified in GSE5058 (Figure 8).

The Affymetrix Human Genome U133 Plus 2.0 Array platform was used for both COPD studies while the SA study was performed using Agilent-014850 Whole Human Genome Microarray 4x44K G4112F. Fluorescence intensity data from individual datasets (raw data) were extracted and processed applying the standard Affimetrix MAS5 algorithm to calculate the fluorescence of the single probes compared to the background. Probes with at least one detected *p* < 0.05 in all three groups were considered for further analysis. Data were then normalized by the median to calculate fold change (FC) expression among groups; only those showing a False Discovery Rate (FDR) ≤ 0.05 in each comparison were considered. As genes are represented on the array platforms by multiple probes spanning different transcript portions, only genes with consistency across probes ≥ 67% were considered for final analysis. The curated gene list of 692 RBPs binding to mRNA (mRBPs) published in a general census of RNA-binding proteins was downloaded from the original file (https://www.nature.com/articles/nrg3813#MOESM25, Supplemetary Information S3) (20) and searched in the final FC files (Figure 1B), producing a list of mRBPs regulated in disease state vs. controls (Figure 1C). The analysis first identified statistically significant genes regardless of fold change value, which were denominated Differentially Expressed Genes (DEG). Then, FCs threshold was set at ≥ |1.5| for identification of genes denominated Significant FC/Differentially Expressed Genes (SDEG) as in standard array analysis.

For the validation analysis, COPD and S data (*n* = 18/18) were extracted from GSE8545 dataset. The GEO2R tool was used for FC data analysis (22).

Generation of Venn diagram analysis for overlapping/unique gene lists was performed using Venny 2.1 (23). Pearson correlation matrix generation was produced using R version 3.6.2. Pearson correlation maps for mRBPs expression changes and heatmaps were generated using tMEV tools v4_9_0.45 (24, 25).

Enrichment for DNA sequence motifs for transcription factors binding sites, according to motif descriptors in the JASPAR database, was identified using PScanChIP (26) with the following parameters: “Organism: Homo Sapiens,” “Assembly: hg38,” “Background: Promoters,” “Descriptors: Jaspar 2018 NR.” Promoter regions have been calculated as the range from +1,500 bp upstream to −500 bp downstream of the transcription start site (TSS) of all the submitted gene list. Only over-represented motifs with *p* ≤ 0.05 were considered.

### Gene Ontology (GO)

GO analysis was performed with Ingenuity Pathway Analysis (IPA) software on microarray probes of RBPs identified as DEG. The significance values for the canonical pathways is calculated by Fisher's exact test right-tailed. The prediction of activation or inhibition of Canonical Pathways was calculated by z-score (27) as follows:

$$\begin{array}{l} z-score=\frac{\sum \left(Upregulated\right)-\;\sum \left(Downregulated\right)}{\sqrt{Ntot}} \end{array}$$

### Statistical Analysis

Statistical analysis was performed using GraphPad Prism 5 (*GraphPad Software Inc*.).

## Results

### Expression Profile of mRBPs in Airway Epithelial Transcriptomic Database of Patients With Stable COPD Compared to Non-smoker and Smoker Control Subjects

**Figure 2:**
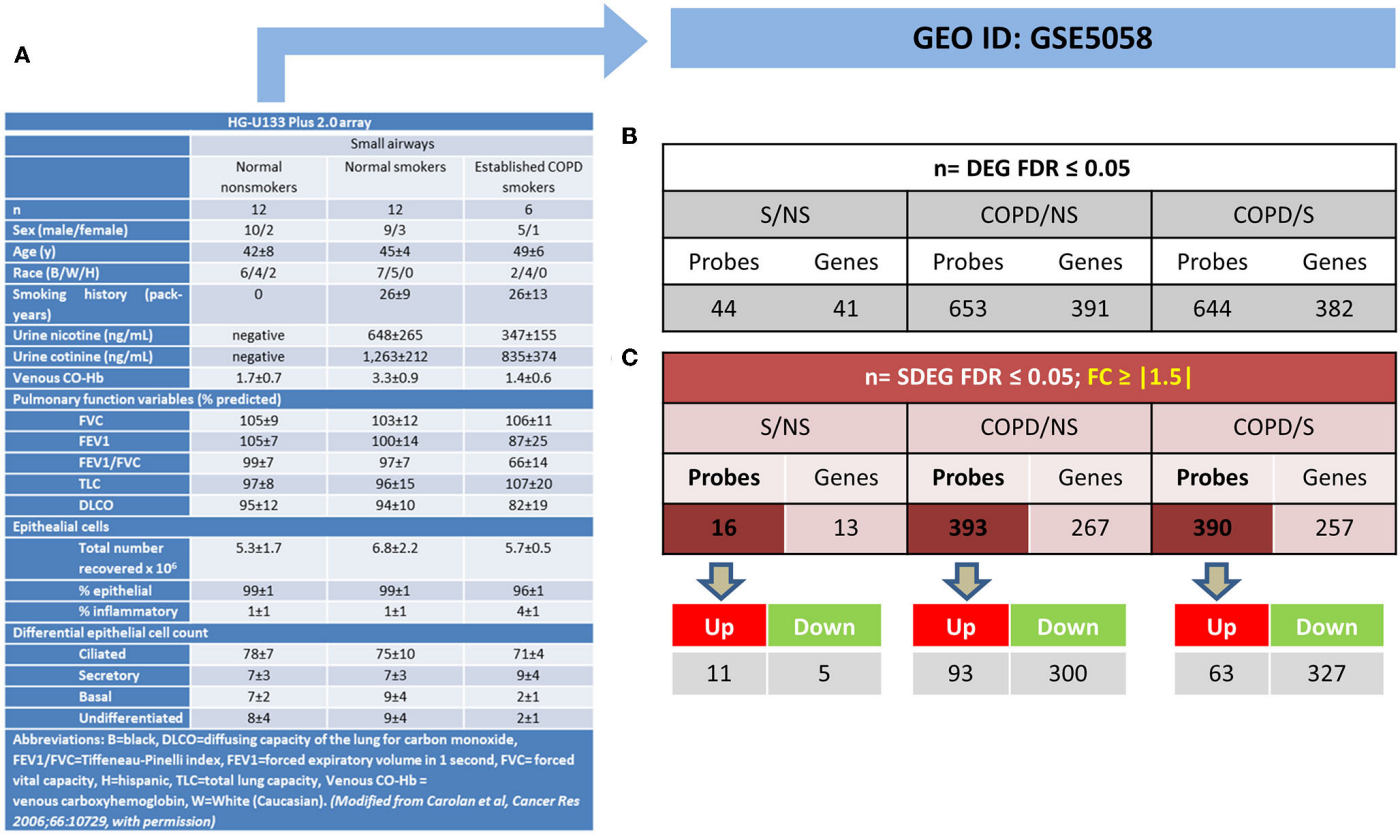
Regulated mRBP expression in Small Airway Epithelial Transcriptomics in stable COPD. **(A)** Clinical and spirometric phenotyping of COPD smoker patients (COPD), non-smokers (NS), and smokers (S) with normal lung function (NLF) as control cohorts providing small airway epithelial cells by bronchial brushings for trascriptomic analysis reported in GEO GSE5058 [modified from (17) with permission] utilized in this study for mRBP expression analysis. **(B,C)** For each gene, multiple probes spanning different gene regions are represented on array platforms. Panels show the numbers of mRBP probes and corresponding genes obtained after FDR filtering (see Figure 1A). **(B)** Statistically significant, Differentially Expressed mRBP Genes, regardless of fold change value (DEG) and **(C)**. Statistically Significant/Differential Expressed mRBP Genes with differential expression set at ≥ |1.5) (SDEG) obtained comparing datasets from S vs. NS, COPD vs. S and vs. NS. Number of Up- or down-regulated SDEG probes are shown below.

**Figure 3:**
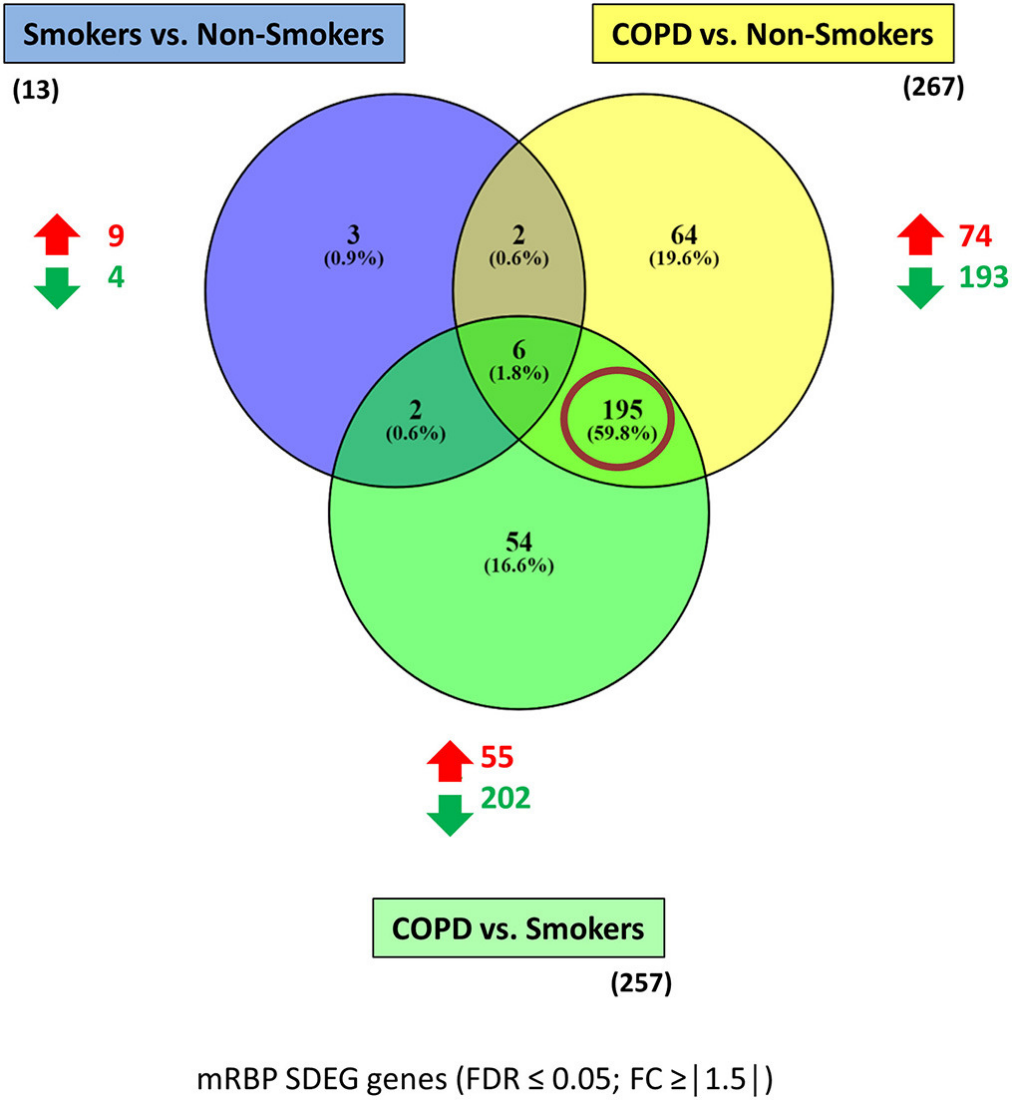
mRBP SDEG genes in small airway epithelium of stable COPD patients vs. non-smoking and smoking control subjects: unique and overlapping regulated expression. Venn diagram generated with Venny 2.1 (23), showing selective and shared mRBPs SDEG genes (FDR ≤ 0.05; FC ≥ |1.5|) among the three comparisons. The number of up-and down-regulated mRBPs for each comparison are shown by red/green arrows; the total number is indicated in parenthesis. Red circle highlights the predominant number of SDEG differentially expressed in COPD, regardless the smoking status of controls.

The mRBPs gene list was searched in the GSE5058 dataset to identify disease-dependent mRBP gene expression in patients with established COPD compared to control groups, clinically phenotyped as shown (Figure 2A). Comparisons of expression levels for the smoking control group vs. non-smoking control group dataset (S/NS) and of COPD patients vs. both NS and S group datasets (COPD/NS, COPD/S) were performed. The number of regulated mRBP genes is reported along with corresponding array probes, which detect different fragments of each gene sequence and generate the fluorescence intensity raw data. We first calculated regulated genes with statistically significant changes regardless of fold change (FC) value, defined as Differentially Expressed Genes (DEG) (Figure 2B). These data revealed an overall greater expression of mRBPs in COPD patients vs. both NS and S control groups, about 9-fold higher than that triggered by smoke exposure alone. The mRBP genes displaying a statistically significant FC value ≥|1.5|, denominated Significant FC/Differentially Expressed Genes (SDEG) were further selected (Figure 2C). Approximately 70% of the mRBP DEG genes were included in this category when COPD samples were compared to both control samples (COPD/NS, COPD/S), while only 30% of S/NS DEG genes were upregulated over this threshold. A small number of genes were excluded from further computing for probe discordance ≥ 67% (*n* = 30/267, 11% for COPD/NS; 10/257 for COPD/S, 3%). The SDEG profiles indicate that the majority of mRBP genes in COPD were downregulated compared to both controls (*n* = 194/267, 73% for COPD/NS; *n* = 202/257, 79% for COPD vs. S) while in S/NS only 30% (*n* = 4/13) were downregulated (Figure 3). Table 1 lists the main functions, published or described in GeneCards (80) for mRBP SDEG with the highest numerical FC value in COPD vs. both control groups.

**Table 1:** Regulated RBPs in COPD vs. normal non-smokers and smokers: selected list with known functions.

**Gene**	**Complete name**	**FC_**COPD VS. S**_**	**FC_**COPD VS. NS**_**	**Main functions**	**References**
FUS	FUS RNA binding protein	−34.63	−37.5	Involved in pre-mRNA splicing and the export of fully processed mRNA to the cytoplasm	(28)
				Maintenance of genomic integrity and mRNA/microRNA processing	(29)
THRAP3	Thyroid hormon receptor associated protein 3	−33.54	−31.58	Enhances the transcriptional activation mediated by PPARG cooperatively with HELZ2	(30)
				Acts as a coactivator of the CLOCK-ARNTL heterodimer	(31)
				Involved in response to DNA damage	(30)
DDX17	DEAD-Box helicase 17	−31.04	−46.27	RNA helicase	(32)
				pre-mRNA splicing, alternative splicing, ribosomal RNA processing and miRNA processing, transcription regulation	(33–36)
				Splicing of mediators of steroid hormone signaling pathway	(37)
				Synergizes with TP53 in the activation of the MDM2 promoter; may also coactivate MDM2 transcription through a TP53-independent pathway	(38–40)
				Coregulates SMAD-dependent transcriptional activity during epithelial-to-mesenchymal transition	(35)
				Plays a role in estrogen and testosterone signaling pathway	(37, 39–41)
				Promotes mRNA degradation mediated by the antiviral zinc-finger protein ZC3HAV1	(42)
SCAF11	SR-Related CTD associated factor 11	−14.4	−12.7	Plays a role in pre-mRNA alternative splicing by regulating spliceosome assembly	(43)
STRAP	Serine/threonine kinase receptor associated protein	−10.8	−8.22	Plays a catalyst role in the assembly of small nuclear ribonucleoproteins (snRNPs), the building blocks of the spliceosome	(44)
				Negatively regulates TGFβ signaling	(44)
				Positively regulates the PDPK1 kinase activity	(44)
RBM14	RNA binding motif protein 14	−7.36	−6.31	General nuclear coactivator, and an RNA splicing modulator. Isoform 1 may function as a nuclear receptor coactivator. Isoform 2, functions as a transcriptional repressor	(45)
				Plays a role in the regulation of DNA virus-mediated innate immune response by assembling into the HDP-RNP complex, a complex that serves as a platform for IRF3 phosphorylation	(46)
BCLAF1	Bcl-2-associated transcription factor 1	−7.32	−4.13	Regulation of apoptosis interacting with BCL2 proteins	(47)
ILF3	Interleukin enhancer binding factor 3	−6.33	−4.61	Forms a heterodimer with ILF2, required for T-cell expression of IL-2	(44)
				Post transcriptional regulation of mRNA binding to poly-U elements and AU-rich elements (AREs) in the 3′-UTR of target mRNA	(48)
				Participates in the innate antiviral response	(49, 50)
				Plays an essential role in the biogenesis of circRNAs	(50)
SFSWAP	Splicing factor SWAP	−5.81	−3.34	Regulates the splicing of fibronectin and CD45 genes	(51)
RBM25	RNA binding motif protein 25	−5.7	−4.16	Regulator of alternative pre-mRNA splicing	(52)
				Involved in apoptotic cell death through the regulation of the apoptotic factor BCL2L1 (proapoptotic isoform S, antiapoptotic isoform L)	(52)
DHX36	DEAH-Box helicase 36	−4.96	−5.31	Enhance the deadenylation and decay of mRNAs with 3′-UTR AU-rich elements (ARE-mRNA)	(53)
				Multifunctional ATP-dependent helicase that unwinds G-quadruplex (G4) structures	(54–57)
				Plays a role in genomic integrity. Converts the G4-RNA structure present in TREC into a double-stranded RNA	(56, 58–62)
				Plays a role in the regulation of cytoplasmic mRNA translation and mRNA stability	(63, 64)
				Plays a role in transcriptional regulation and post-transcriptional regulation	(54, 65, 66)
HNRNPA2B1	Heterogeneous nuclear ribonucleoprotein A2/B1	−4.91	−4.63	Associates with nascent pre-mRNAs, packaging them into hnRNP particles and drive them into transcription, pre-mRNA processing, RNA nuclear export, subcellular location, mRNA translation and stability of mature mRNAs.	(67)
				Involved in transport of specific mRNAs to the cytoplasm in oligodendrocytes and neurons recognizing binding the A2RE or the A2RE11 sequence motifs present on some mRNAs.	(68)
				Specifically binds single-stranded telomeric DNA sequences, protecting telomeric DNA repeat against endonuclease digestion	(69)
				Involved in the transport of HIV-1 genomic RNA out of the nucleus, to the MTOC, and then from the MTOC to the cytoplasm: acts by specifically recognizing and binding the A2RE sequence motifs present on HIV-1 genomic RNA.	(69)
CCAR1	Cell division cycle and apoptosis regulator 1	−2.79	−2.93	Plays a role in cell cycle progression and/or cell proliferation	(70, 71)
				p53 coactivator	(72)
NR0B1	Nuclear receptor subfamily 0 Group B member 1	1.57	3.31	Acts as a dominant-negative regulator of transcription which is mediated by the retinoic acid receptor	(73)
				Functions as an anti-testis gene by acting antagonistically to Sry	(73)
				Plays a role in the development of the embryo and in the maintenance of embryonic stem cell pluripotency	(73)
RBPMS	RNA-Binding protein with multiple splicing	1.77	1.4	pre-mRNA maturation (binds to poly(A) RNA)	(74, 75)
				Required to increase TGFB1/Smad-mediated transactivation	(75)
HDLBP	High density lipoprotein binding protein	1.91	2.53	Regulates excess cholesterol levels in cells	(76)
				Induces heterochromatin formation	(76)
MATR3	Matrin 3	2	3.3	Plays a role in the regulation of DNA virus-mediated innate immune response by assembling into the HDP-RNP complex, a complex that serves as a platform for IRF3	(46)
DHX30	DExH-Box helicase 30	2.18	2.45	Assembly of the mitochondrial large ribosomal subunit	(77, 78)
				Required for optimal function of the zinc-finger antiviral protein ZC3HAV1	(79)
				Involved in nervous system development differentiation	(79)

*A2RE, 21 nucleotide hnRNP A2 response element; A2RE11, 11-nucleotide subsegment of the A2RE; ARE, AU Rich Elements; ARNTL, Aryl Hydrocarbon Receptor Nuclear Translocator Like; circRNA, circular RNA; CLOCK, Circadian Locomoter Output Cycles Protein Kaput; HELZ2, Helicase With Zinc Finger 2; hnRNP, heteronuclear ribonucleoprotein; IL-2, Interleukin2; IRF3, Interferon Regulatory Factor 3; MDM2, Proto-oncogene MDM2; miRNA, micro RNA; MTOC, microtubule organizing center; NONO, Non-POU Domain Containing Octamer Binding; PDPK1, 3-Phosphoinositide Dependent Protein Kinase 1; rRNA, ribosomal RNA; SMAD, small mother against decantaplegic; TGFβ, Transforming Growth Factor β; TP53, Tumor Protein 53; TREC, telomerase RNA template component; ZC3HAV1, Zinc Finger CCCH-Type Containing Antiviral 1*.

The three gene groups (NS/S, COPD/NS, COPD/S) were intersected to identify both unique and overlapping mRBP expression profiles. As shown in the Venn's diagram (Figure 3), 195 SDEG genes are shared between the COPD patients vs. both NS and S groups, pointing at a distinctive mRBP signature driven by COPD beyond the active exposure to cigarette smoke.

**Figure 4:**
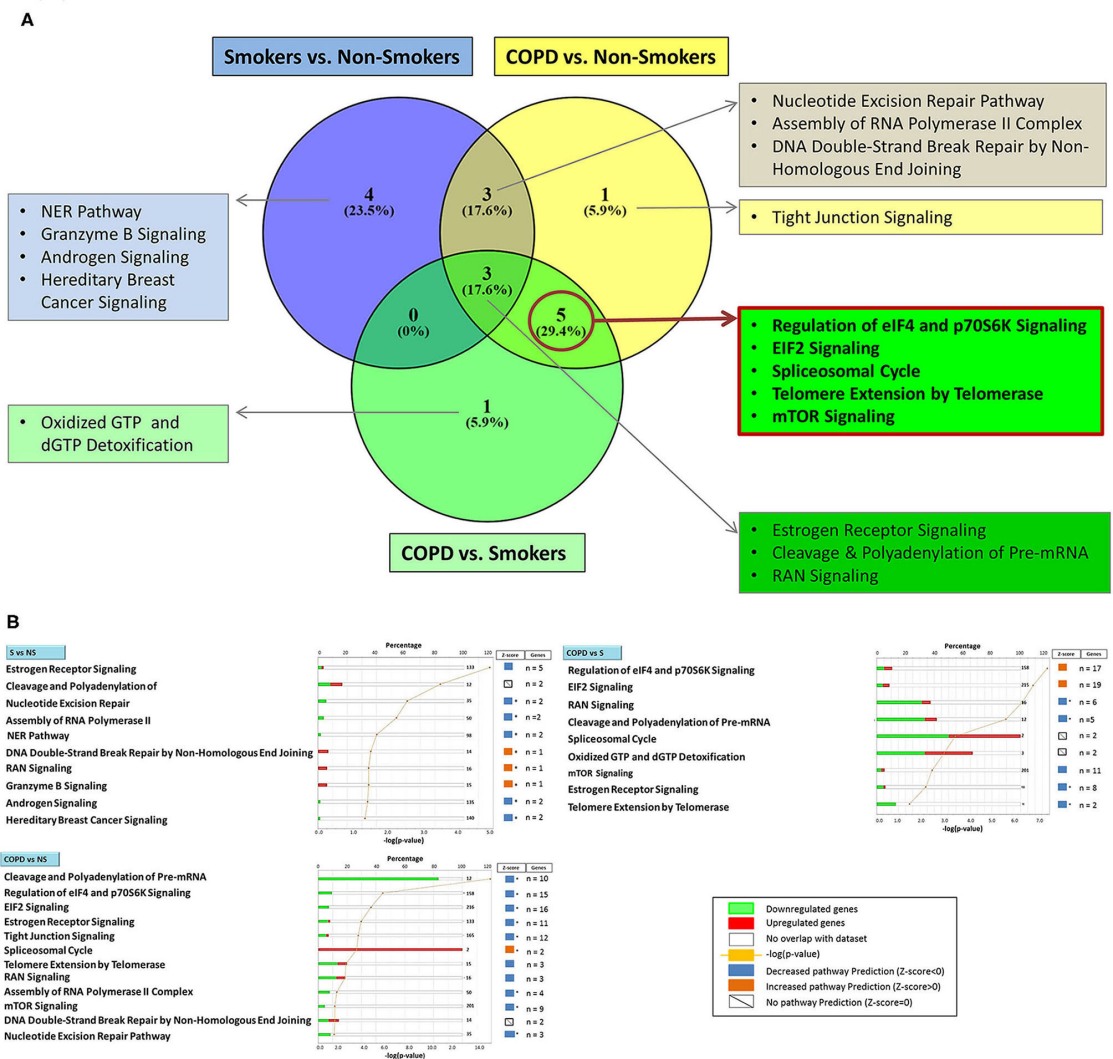
Genome Ontology analysis of mRBPs expression in small airway epithelium of stable COPD patients vs. non-smoking and smoking control subjects: involvement in established COPD pathogenic pathways. Ingenuity Pathway Analysis (IPA) of mRBP in COPD GSE5058 Dataset. **(A)** Venn diagram showing selective and shared canonical pathways among the group comparisons, calculated by Ingenuity Pathway Analysis (IPA) on DEGs for increased statistical power and listed as in Figure 3. In evidence (red lined rectangle) the pathways enriched in COPD vs. both NS and S control groups. **(B)** List of canonical pathways identified for each group comparisons. For each pathway, bargraphs indicate the number of RBP genes associated with the pathway (total number listed at the end of the bar) that were found up (red)- or down (green)-regulated in the dataset; z-score (on the right) predicts pathway repression (blue) or activation (orange) given by the expression profile (see Methods); far right: genes n = indicates number of regulated mRBP genes involved for each pathway (also listed in Table 2). * z-score ≥ |1|.

Using the larger DEG lists, the three gene sets were then analyzed by IPA software probing changes in the categories of canonical pathways and intersected, (Figure 4) as done for the SDEG gene groups. Five out of eight canonical pathways impacted by COPD were shared by comparisons to NS and S groups (Figure 4A). Calculation of the z-score parameter yielded a predictive assessment of the downstream effect - activation or inactivation - exerted by the identified RBP profile on the metabolic pathways (Figure 4B). Table 2 lists the regulated mRBPs impacting upon the canonical pathways shown in Figure 4B.

**Table 2:** GO analysis by IPA indicating canonical pathways in which RBP enrichment is significant for each comparison, with predicted functional outcome indicated by z-score (See Methods for details), and RBP molecules involved.

**2A. S/NS**				
**Ingenuity canonical pathways**	***p*****-value**	**z-score**	**Prediction**	**Molecules**
Estrogen receptor signaling	1.38E-05	−0.44	Inhibition	PRKDC,NR0B1,SPEN,POLR2H, POLR2L
Cleavage and polyadenylation of Pre-mRNA	3.47E-04	0	0	CPSF2,CPSF6
Nucleotide excision repair pathway	3.02E-03	−1.41	Inhibition	POLR2H,POLR2L
Assembly of RNA polymerase II complex	6.03E-03	−1.41	Inhibition	POLR2H,POLR2L
NER pathway	2.19E-02	−1.41	Inhibition	POLR2H,POLR2L
DNA double-strand break repair by non-homologous end joining	3.24E-02	1	Activation	PRKDC
RAN signaling	3.63E-02	1	Activation	TNPO1
Granzyme B signaling	3.63E-02	1	Activation	PRKDC
Androgen signaling	3.98E-02	−1.41	Inhibition	POLR2H,POLR2L
Hereditary breast cancer signaling	4.68E-02	−1.41	Inhibition	POLR2H,POLR2L
**2B. COPD/NS**				
Cleavage and polyadenylation of pre-mRNA	2E-15	−3.16	Inhibition	PAPOLA,CPSF2,CPSF6,CSTF1,NUDT21,CPSF1,CSTF2,CPSF3, CSTF3,CPSF4
**Regulation of eIF4 and p70S6K signaling**	2.88E-06	−3.87	Inhibition	EIF2B4,PAIP2,EIF3E,EIF4G1,EIF2B2,EIF4E,EIF3M,EIF3G,EIF1, EIF3B,EIF3A,EIF2B1, EIF3L,EIF1AX,EIF3K
**EIF2 signaling**	3.02E-05	−4	Inhibition	EIF2B4,EIF3E,EIF4G1,EIF2B2,EIF4E,EIF3,EIF3G,PTBP1,EIF1,EIF3, HNRNPA1,EIF2B,EIF3A,EIF3L,EIF1AX,EIF3K
Estrogen receptor signaling	0.000209	−2.11	Inhibition	PRKDC,DDX5,THRAP3,SPEN,NR0B1,POLR2H,GTF2F1,HNRNPD, RBFOX2,POLR2K,POLR2L
Tight junction signaling	0.000363	−1.73	Inhibition	CPSF2,CPSF6,CSTF1,NUDT21,CPSF1,CSTF2,YBX3,CPSF3, SYMPK,SAFB,CSTF3,CPSF4
**Spliceosomal cycle**	0.000501	1.41	Activation	U2AF1/U2AF1L5,U2AF2
**Telomere extension by telomerase**	0.004169	−0.57	Inhibition	HNRNPA1,XRCC6,HNRNPA2B1
RAN signaling	0.005129	−0.57	Inhibition	KPNB1,RANBP2,TNPO1
Assembly of RNA polymerase II complex	0.025704	−2	Inhibition	POLR2H,GTF2F1,POLR2K,POLR2L
**mTOR signaling**	0.038019	−3	Inhibition	EIF3G,EIF3B,EIF3A,EIF3E,EIF4G1,EIF4E,EIF3L,EIF3M,EIF3K
**2C. COPD/S**				
**Regulation of eIF4 and p70S6K signaling**	5.75E-08	0.24	Activation	EIF2B4,EIF4G3,PAIP2,EIF3E,EIF4G1,EIF2B2,EIF4E,EIF3M,EIF3G, EIF1,EIF3B,PAIP1,EIF3A,EIF2B5,EIF1AX,EIF3L,EIF3K
**EIF2 signaling**	2.24E-07	0.22	Activation	EIF2B4,EIF4G3,EIF3E,EIF4G1,EIF2B2,EIF4E,EIF3M,EIF3G,PTBP1, EIF1,EIF3B,HNRNPA1,EIF5,PAIP1,EIF3A,EIF2B5,EIF1AX,EIF3,EIF3K
RAN signaling	6.46E-07	−1.63	Inhibition	KPNB1,RANBP2,TNPO1,RAN,XPO1,IPO5
Cleavage and polyadenylation of pre-mRNA	3.16E-06	−1.34	Inhibition	PAPOLA,CPSF2,CSTF1,NUDT21,CSTF3
**Spliceosomal cycle**	0.000468	0	0	U2AF1/U2AF1L5,U2AF2
Oxidized GTP and dGTP detoxification	0.00138	0	0	DDX6,RUVBL2
**mTOR signaling**	0.004365	−0.30	Inhibition	EIF3G,EIF3B,EIF3A,EIF4G3,EIF3,EIF4G1,EIF3L,EIF4E,EIF3M, EIF4B,EIF3K
Estrogen receptor signaling	0.008318	−1.41	Inhibition	PRKDC,THRAP3,NR0B1,SPEN,HNRNPD,GTF2F1,POLR2K,POLR2
**Telomere extension by telomerase**	0.040738	−1.41	Inhibition	HNRNPA1,HNRNPA2B1

*In bold are the pathways associated with COPD vs. both controls*.

**Figure 5:**
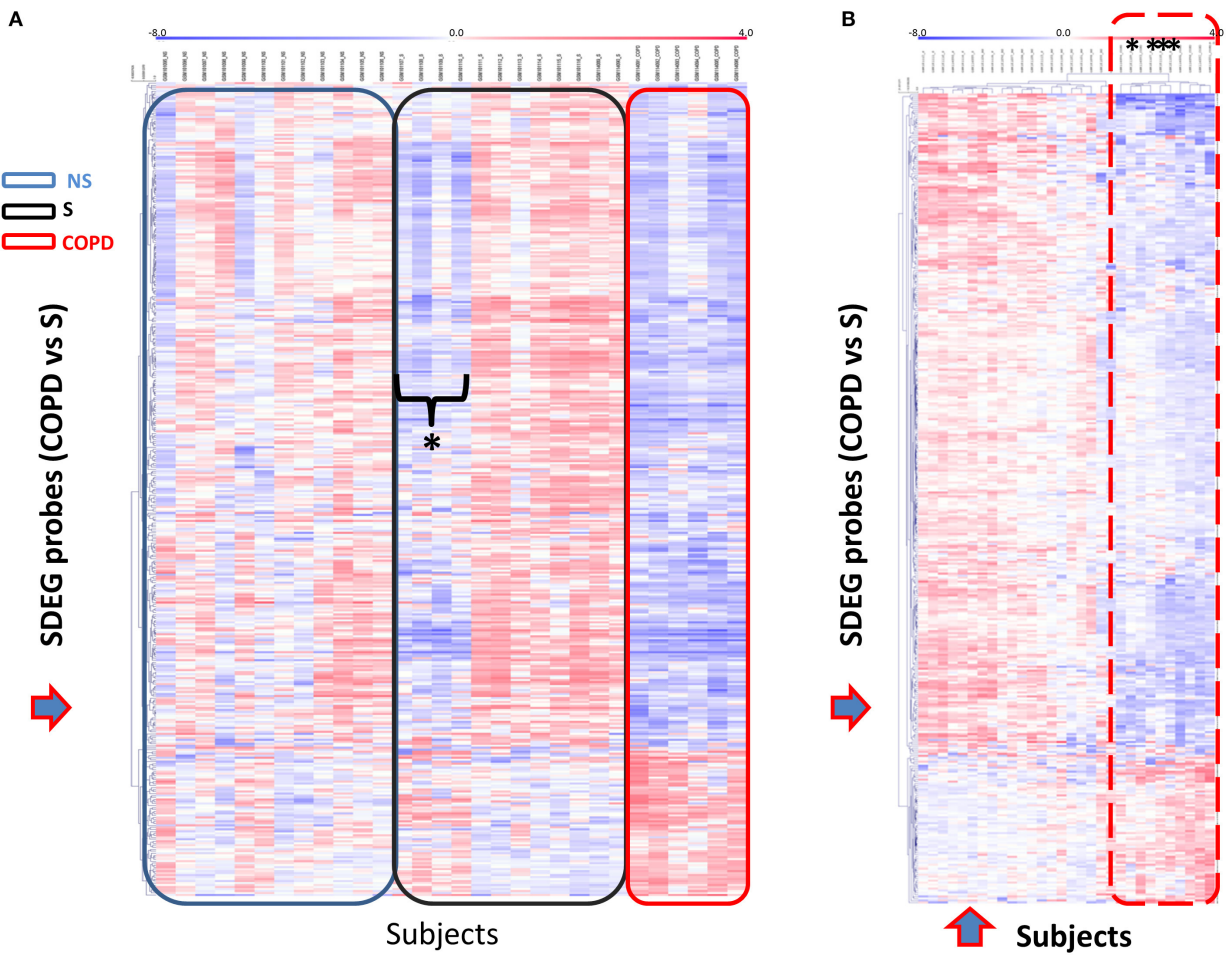
Unsupervised gene cluster analysis across the individual samples from GSE5058 dataset identifies selective global mRBPs repression in COPD patients shared by a subset of smoker controls. **(A)** Unsupervised clustering analysis applied to (blue arrow) SDEG probe list identified in COPD/S (*n* = 409). Heatmap shows SDEG probes' fluorescence intensity value (blue < 0, reduced: red > 0, increased). The data were normalized on the median and log_2_-trasformed for relative fold changes (See Methods). **(B)** Unsupervised clustering analysis applied to both SDEG probe list and individual samples (blue arrows). Asterisks indicate the SDEG profiles of four smokers with NLF, clustering with those of COPD patients indicated by the dotted line.

We then selected the gene set obtained for the comparison COPD/S (*n* = 409 SDEG probes) to perform an unsupervised clustering analysis of each subject's mRBP expression profile for the three groups (NS, S, COPD), using Pearson's hierarchical clusters/complete linkage method. Results were represented in a heatmap generated with the T-MeV software (Figure 5) (24, 25). As expected, the heatmap clearly showed how the expression of mRBPs appears predominantely diminished compared to NS and most of S subjects; interestingly, it also identified in the S group a subset of four subjects displaying an expression profile highly homologous to the one identified in COPD patients (Figure 5A). This similarity was confirmed by performing unsupervised cluster analysis for both genes and subjects using an Euclidean distance metrics (Figure 5B). This analysis confirmed that the RBP expression profile of this S subgroup indeed clustered with the samples from COPD patients.

**Figure 6:**
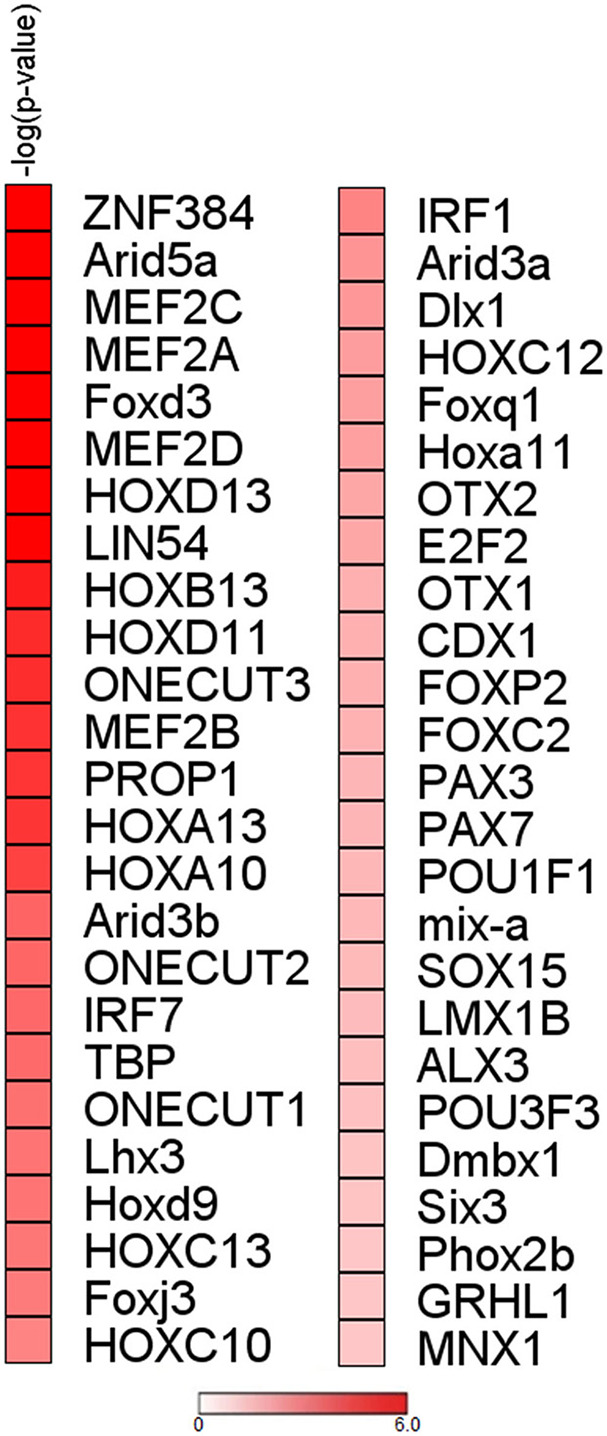
Analysis of upstream regions of mRBP genes regulated in COPD/S identifies enrichment of transcription factors binding sites for downregulated genes. Enrichment analysis of transcription factor binding sites for COPD/S SDEG gene list (*n* = 257) within the promoter regions orderd by -log(*p*-value). Promoter regions have been calculated as the range from +1,500 bp upstream to −500 bp downstream of the gene transcription start site (TSS). Only over-represented motifs with *p* ≤ 0.05 were considered.

To initiate mechanistic understanding of predominant RBP downregulation in subjects with stable COPD and in selected smokers with normal lung function, we conducted for the RBP gene set obtained for COPD/S a search for transcription factor (TF) binding motif within promoter regions. Significant binding site enrichment for several TFs (Figure 6, listed in Supplementary Table 1) was exclusively found for the genes downregulated in their expression.

**Figure 7:**
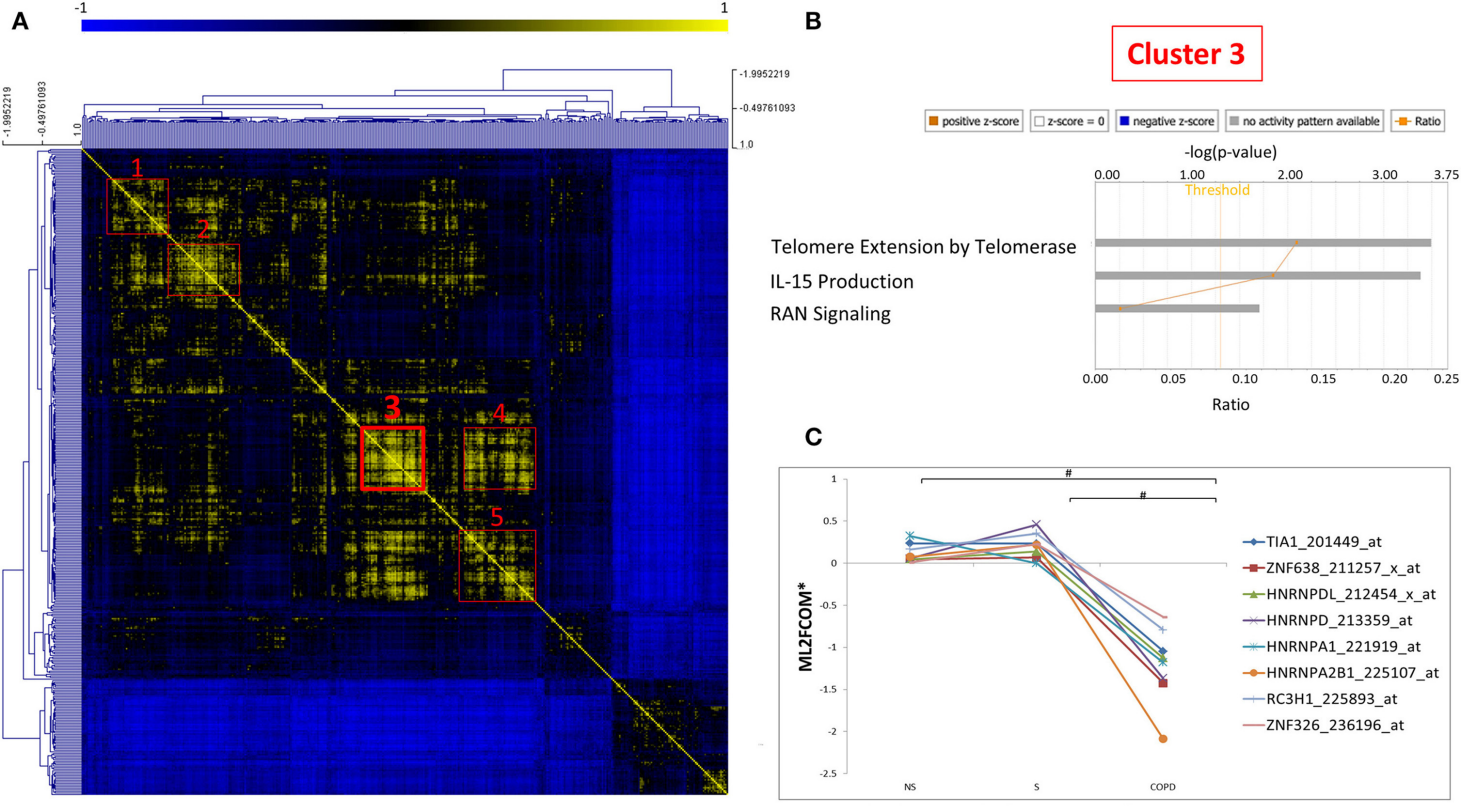
Correlation map identifies five clusters of coexpressed RBPs in COPD. **(A)** Pearson Correlation maps of SDEG probe list (*n* = 409 in COPD/S) across all samples, with *R* value set as (*r* ≥ 0.7) identifies at least five clusters of coexpressed mRBPs (red squares). **(B)** Representative GO analysis of cluster 3 (*n* = 42 SDEG) (full GO cluster analysis in Table 3). **(C)** Representative expression profile of selected mRBPs coexpressed in cluster 3 (full cluster expression profiles in Supplementary Table 2). ^#^*p* ≤ 0.05. *Mean of Log_2_ (fold change over median).

As mRBPs exert their function by dinamically assembling in RNP complexes, the same gene dataset (SDEG in COPD/S) was then searched for RBPs with correlated expression, which may indicate disease-driven, coordinated target regulation (20). Pearson correlation map showed at least five highly correlated mRBP clusters (*r* ≥ 0.70) (Figure 7A). Clusters of RBP gene lists and their FC values between COPD and both S/NS control populations are listed in Supplementary Table 2. Canonical pathway analysis indicated that RBP genes in clusters commonly impacted upon RAN signaling and Telomere Extension by Telomerase, along with more cluster- restricted influence on other pathways, such as inhibition of ARE-mediated mRNA degradation and IL-15 expression (Table 3). In particular, cluster 3 included 40 genes, among which was included *HNRNPD*, coding for the RBP AUF-1 that we previously identified as repressed in small airway epithelium in airway biopsies of an independent subject cohort of COPD patients compared to smoker controls (16). For cluster 3, IPA analysis identified enrichment of genes involved in RAN signaling, telomere extension, IL-15 expression (Figure 7B). Figure 7C shows normalized fold changes across the three groups for eight representative mRBPs, including *HNRNPD*, contained in cluster 3.

**Table 3:** Canonical Pathways identified by IPA analysis of mRBPs clusters 1 to 5 as shown in Figure 7.

**Ingenuity canonical pathways**		
**Cluster 1**	***p*****-value**	**Molecules**
RAN signaling	2.24E-02	TNPO1
Apelin muscle signaling pathway	2.51E-02	TFAM
**Cluster 2**	***p*****-value**	**Molecules**
RAN Signaling	1.20E-02	RANBP2
Pyrimidine ribonucleotides interconversion	2.95E-02	DDX3X
Pyrimidine ribonucleotides *de novo* biosynthesis	3.09E-02	DDX3X
**Cluster 3**	***p*****-value**	**Molecules**
Telomere extension by telomerase	3.24E-04	HNRNPA1,HNRNPA2B1
RAN signaling	4.17E-04	RANBP2,TNPO1
IL-15 production	2.00E-02	CLK1,CLK4
**Cluster 4**	***p*****-value**	**Molecules**
RAN signaling	1.48E-05	RANBP2,TNPO1,XPO1
Telomere extension by telomerase	8.32E-04	HNRNPA1,HNRNPA2B1
Cleavage and polyadenylation of pre-mRNA	3.39E-02	NUDT21
IL-15 production	4.68E-02	CLK1,CLK4
Inhibition of ARE-Mediated mRNA degradation pathway	4.79E-02	DCP2,TIA1
**Cluster 5**	***p*****-value**	**Molecules**
Telomere extension by telomerase	3.31E-04	HNRNPA1,HNRNPA2B1
Ran signaling	4.27E-04	RANBP2,XPO1
Inhibition of ARE-mediated mRNA degradation pathway	2.04E-02	DCP2,TIA1
Cleavage and polyadenylation of pre-mRNA	2.14E-02	NUDT21

**Figure 8:**
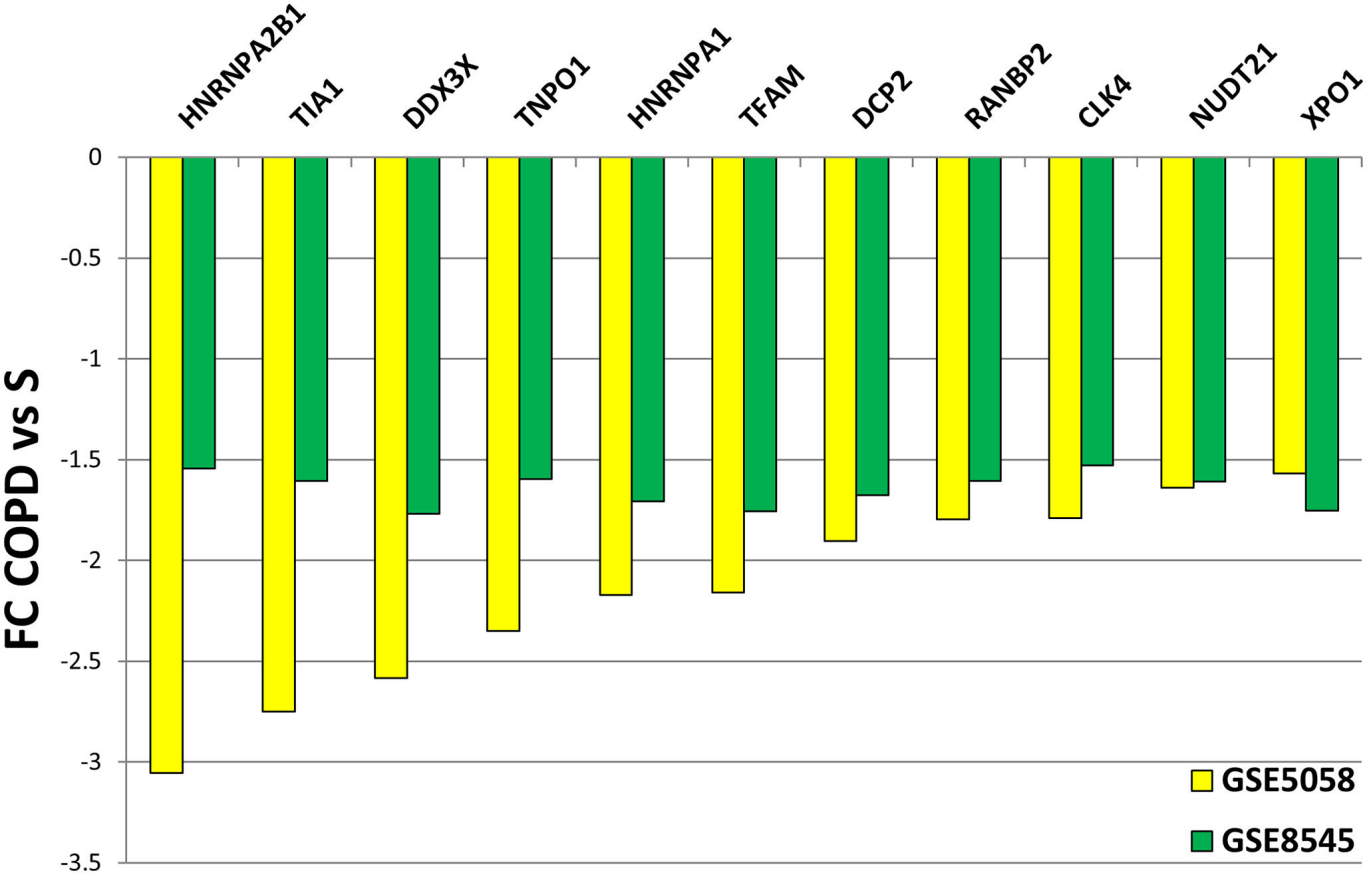
Validation of differential expression for RBP genes in GSE8545 COPD/S dataset. Comparison of FC values for COPD/S mRBP genes between GSE5058 (test dataset) and GSE8545 (validation dataset; See Methods) for the RBP cluster genes (listed in Table 3) identified by IPA as having significant impact on canonical pathways.

We then submitted the mRBP profile obtained in the COPS/S, used also to identify gene clusters, to validation in an independent transcriptomic dataset of small airway epithelium obtained from bronchial brushing of NS, S and COPD (GOLD stages I or II) [*n* = 18 each cohort, deposited in public GEO repository as GSE 8545 (21). Analysis of COPD/S in GSE 8545 largely confirmed the global downregulation of RBP in COPD/S, with 56% of probe sets downregulated with an FC ≤ - 1.5 (217/390) vs. 80% (327/390) downregulated in GSE5058. Importantly, the comparison reproduced differential expression for all RBP cluster genes (listed in Table 3) identified by IPA as having significant impact on canonical pathways (Figure 8).

### Expression Profile of mRBPs in Bronchial Epithelium of Patients With Severe Asthma Compared to Control Subjects

Transcriptomic data in airway epithelium from bronchial brushing of patients with severe asthma (SA) and healthy controls (HCs) were searched for mRBP expression using the same methodology (Figure 1). Clinical characteristics of the original study, from which we extracted GEO data only for SA and HC groups, are shown in Supplementary Table 3. We randomly extracted from the GSE63142 dataset (19) the same number of patients of the GSE5058 COPD dataset (*n* = 6 SA, *n* = 12 HCs). Only 30 probes (corresponding to 29 genes) were differentially expressed (DEG) in SA vs. HCs, but none of the DEG genes changed by at least 50% compared with HCs, thus none was categorized as SDEG (FDR ≤ 0.05; FC ≥ |1.5|). We then extended the search to the entire number of subjects of the datasets (*n* = 56 SA and 27 HCs) and identified as DEG 68 gene probes corresponding to 62 genes; as for the previous analysis, none of the DEG was categorizable as SDEG (genes listed in Supplementary Table 4).

## Discussion

In the present study, global changes in mRBP gene expression in human airway epithelium were evaluated, for the first time, using public gene array databases derived from bronchial brushings of patients affected by two major chronic lung inflammatory diseases, COPD and severe asthma, and their relative control subjects. We report that significant changes were largely exclusive to samples from COPD and that they were mostly due to downregulated mRBP expression, a feature that was found also in a subset of control smokers. These changes were associated with the occurrence of several clusters of co-regulated mRBPs, and they impacted relevant pathways deemed pathogenic for COPD.

We recently identified in a GEO database of airway epithelial cells from stable COPD patients a significant loss of the RBP AUF-1 compared to smoker controls, matching the loss we identified at protein level in airway samples of patients with stable COPD compared to normal smokers (16). The present study was undertaken on this basis as a broader investigation of epithelial RBP patterns comparing two lung diseases characterized by chronic inflammation and oxidative stress – COPD and SA - since preclinical studies clearly identified the role of this class of posttranscriptional regulators in these pathological processes (14, 81, 82), yet they are scarcely described in human disease driven by them. This proof-of concept study could serve as springboard for studies focused on specific PTGR pathways and molecular species exploitable as phenotype/endotype disease biomarker or for therapeutic targetability.

To generate the list of mRBPs we used for this study, Gerstberger et al. (20) searched the human genome for protein-coding genes bearing RBDs using specific statistical probability models and futher manual curation, which led to a final census of 1,542 RBPs. Strikingly, the biological functions of a third of these proteins is unknown or minimally defined, at least in human disease; some of these - such as DEAD/DEAH box helicases – were found to be significantly regulated in airway epithelium of COPD patients by our analysis.

In our study the DEAD-box RNA helicase, DDX17 was in fact among the top downregulated mRBP in COPD; its role is not yet defined in this disease. DDX17 is a nucleocytoplasmic shuttling factor that functions as RNA helicase and is involved in transcription, splicing and miRNA processing. Several studies indicate its involvement in antiviral responses: in a recent study, a significant decrease in DDX17 was found in transcriptional signature to vaccination to H1N1 influenza virus in human subjects, which correlated with antibody titter and IFN-γ production by T-cells (83). Upregulated DDX17 expression has been reported to be associated with resistance to the tyrosine kinase inhibitor drug, gefitinib in non-small cell lung cancer (NSCLC) cells (84). We also found as significantly downregulated the 3′-5′ DEAH-box helicase DHX36, also known as RHAU (RNA helicase associated with AU-rich element) (58). In addition to regulating the transport and half-life of ARE-bearing mRNAs, it has a role in the mechanisms of preservation of genome integrity and in the maintenance of telomeres. In particular, DHX36 assists the activity of the TERT (Telomerase Reverse Transcriptase) enzyme (54, 58–62). As helicase, DHX36 unwinds parallel G-quadruplex structures formed in DNA and RNA. Interestingly, a recent study indicates that ablation of DHX36 results in increased SG formation and protein kinase R (PKR/EIF2AK2) phosphorylation, indicating that DHX36 is involved in resolution of cellular stress at the level of SG (85). Moreover, in rat alveolar epithelial cells DHX36 downregulates epithelial sodium channel (ENaC) mRNA stability by binding with T-cell internal antigen-1 related protein (TIAR-1) to the transcript 3′-UTR (86).

The results from the study in COPD revealed for the first time a significant, global downregulation of mRBPs in cells from COPD patients compared to controls; interestingly, a pattern very similar was found in samples from four out of 12 smoker controls with normal lung function, as confirmed by cluster analysis. This data suggest that this expression profile could indicate subjects at higher risk related to smoke, or with disease at preclinical stage. It would be important to understand, upon validation on larger datasets, the specific mechanisms and epithelial responses affected by this mRBP profile, and whether it leads to molecular changes underpinning a specific clinical disease phenotype.

To this end, a search for common transcriptional control and global pathway analysis may assist in directing further studies on a scale larger than single-gene analysis, in particular when exploring relatively uncharted pathways.

Of note, significant enrichment for TF binding motifs in COPD/S-regulated genes was found only for downregulated RBP, further supporting a biological relevance of this global change, possibly representing a coordinated shift in response to pathogenic triggers. Overall, the TFs putatively binding to the DNA motifs identified with highest enrichment, such as the MEF2 TF family, have pleiotropic functions in cell cycle, cell differentiation, proliferation, apoptosis (87); Interestingly, ARID5A acts both as TF and as RBP according to nuclear or cytoplasmic localization (88); FoxD3 acts as tumor suppressor in lung cancer (89) while HOX genes are overexpressed in non-small cell lung cancer and postulated to act as oncogenes (90).

In our genome ontology analysis (Table 2), some of the pathways significantly affected by the changes in mRBPs are already recognized as impacted by the pathogenetic process in COPD, such as the expression/activity of the telomerase enzyme and the signaling coordinated by the kinase mTOR (Mammalian Target Of Rapamycin) (91–94); others may indicate so far under recognized disease determinants.

Telomerase is an enzyme complex that reverse-transcribes an integral RNA template in order to add short DNA repeats at the 3′-ends of telomeres. In our study, the mRBPs HnRNPA1 and HnRNPA2B were found as downregulated SDEG in COPD and this profile was predicted by IPA to inhibit telomere extension by telomerase. Early studies established that hnRNP-A1, hnRNP-A2, and hnRNP-B1 proteins can interact with telomeres and are products of two different genes (*HNRNPA1* and *HNRNPA2B1*) but display similar structures (two RRMs and four RGG motifs in each)(95). The mRBP hnRNP A1 is the best characterized and found to be associated with human telomeres *in vivo* (96); depletion of hnRNP A/B proteins in human embryonic kidney 293 cell extracts greatly reduced telomerase activity, which was rescued by addition of recombinant hnRNP A1 (96). Recently, a large study conducted in a group of 576 patient with moderate-to-severe COPD found a significant relationship of absolute telomere length, measured by PCR in DNA from peripheral blood samples, with several clinical parameters such as quality of life, number of exacerbations, and mortality (97). These evidence suggest that shorter leukocyte telomere lengths could be evaluated as a biomarker for clinical outcomes in COPD. Furthermore, these two RBPs were coexpressed in cluster 3 (Figure 7), along with another known TERT-regulating factor, AUF-1 (98), which we initially documented as downregulated in COPD patients vs. controls by immunohistochemistry (16). AUF-1 positively regulates TERT1 at promoter level, but its loss may impact cellular senescence also by concomitant lack of its destabilization function for cell-cycle checkpoint mRNAs (98). Coexpression is often found among RBPs that participate to common posttranscriptional pathways; therefore, the novel mRBP clusters we identified (Figure 7 and Supplementary Table 2) can be used as starting point to infer mRBP putative regulatory roles and identify coordinated expression of targets (20). Taken together, depletion in COPD of RBPs that are crucial for telomere length, as suggested by our findings, may have a role in shaping several COPD clinical outcomes by impacting this function also in airway epithelium.

In our pathway analysis, signaling of eukariotic initiation factors (eIF) was negatively impacted by downregulation of several eIFs, an effect which was also linked to a negative influence on mTOR signaling. The expression and functions of eukariotic initiation factors (eIF) are generally regulated in aging, transformation, and growth arrest and are critically hampered in cancer and during pathogenic stress conditions (99). Acceleration of cellular aging driven by oxidative stress in COPD lung is characterized by activation of PI3K (phosphoinositide-3-kinase) and mTOR signaling, through oxidation of tyrosine phosphatases such as and SHIP-1 (SH2-containing inositol-59-phosphatase-1) and PTEN (phosphatase tensin homolog) (100). The activity of eIF4E-binding protein(BP)1/eIF4E pathway is required for mTOR-dependent G1-phase progression (101), in particular it mediates mTORC1-dependent increased expression of cyclin D1 (102). In cancer cells, decreased expression and functions of eIFs leads to inhibition of global translation and enhancement of translation of subsets of mRNAs by alternative mechanisms (99). Therefore, pathway analysis of COPD-related mRBP patterns strongly suggests a key role for altered translational regulation as a pathogenic mechanism by which oxidative stress alters specific protein levels and cellular functions. A more specific understanding of dysregulation of mRNA translation could uncover new strategies to diagnose and treat at least some forms of chronic inflammation and possibly indicate mechanisms linking COPD to lung cancer development.

Cytokines, chemokines and proinflammatory molecules secreted by senescent cells are collectively described as the senescence-associated secretory phenotype (SASP) (103). Multiple RBPs, such as Human antigen (Hu)R, AUF-1, tristetraprolin (TTP) have been shown to regulate several of the inflammatory transcripts that take also part to the SARP phenotype (12, 81, 104). In particular, CCL2, CXCL1 and IL-6 were identified among others as HuR-associated and regulated transcripts in human airway epithelial BEAS-2B cells stimulated with TNFα plus IFNγ. However, levels of TTP expression appear unchanged in airway samples from COPD patients and smoker controls as well as in epithelial *in vitro* models of cigarette smoke challenge, and there are conflicting evidence regarding airway epithelial HuR expression in COPD (16, 105, 106). The documented loss of epithelial AUF-1 may contribute also to SASP, besides impacting on cellular senescence, through loss of its mRNA decay-promoting function for several of its factors, such as IL-1β, TNFα, IL-6, CXCL1, VEGF (104). It is noteworthy that in our correlation analysis of COPD-regulated mRBPs, expression of T-cell intracellular antigen (TIA)-1, a critical translational repressor of TNFα (107), clusters with that of AUF-1 (Figure 7C).

Lastly, the pathway impacted by COPD-associated RBP expression in all clusters is Ras-related nuclear protein (Ran) signaling. Ran controls nucleo-cytoplasmic protein transport through the nuclear pore complex and regulates cell cycle progression (108, 109). As regulators of RNA throughout their lifespan, numerous RBPs undertake regulated nucleocytoplasmic shuttling in complex with their targets; key RBPs, such as HuR and HNRNPA1, are known to bind to transportin-1 (110). Conversely, several factors involved in Ran-mediated nucleo-cytoplasmic transport system, including transportin 1, are controlled by RBPs such as HuR (111) and by posttranscriptional control at large. As disruption of Ran expression and function is linked to most cancers, including NSCLC (112, 113), this data suggest that the impact of RBP expression changes on Ran pathway may contribute to increased risk of cancer development in a subset of COPD patients.

Unexpectedly, no significant differential mRBP expression was found when applying the mRBP list search to the database derived from bronchial brushings of SA patients vs. healthy controls. Triggers and inflammatory features of bronchial asthma have distinct features from those of COPD, however in severe asthma the inflammatory pattern is less divergent: increased oxidative stress and a poor response to corticosteroids are SA features common to COPD, especially in SA subjects who are smokers (114). Moreover, global alterations in airway epithelial-derived miRNA and polysome-bound mRNAs have been reported in other smaller SA patient cohorts compared to HC, indicating the occurrence of alteration in posttranscriptional pathways (115). Furthermore, a recent study reports that in primary airway epithelial cells harvested by bronchial brushing in SA patients, IL-17A in combination with TNFα prevents the cytoplasmic translocation of the RBP TIAR. Nuclear sequestration of TIAR halted the cytoplasmic binding and translational repression of its targets, IL-6 and CXCL8 mRNAs and correlated with corticosteroid insensitivity, *in vivo* neutrophilic inflammation and the post-bronchodilator FEV1 of SA patients (116). Posttranscriptional changes in asthma could be driven by a more diverse pattern of molecular species and mechanisms and changes in mRBPs localization and binding, rather that expression, may be predominant. This underscores the need to implement studies probing RBP posttranslational modifications in human airway samples or cell lines and to characterize the RBP interactome and understand its regulation of disease-related epithelial responses. More studies will be necessary to validate this significant negative finding in SA also using additional airway epithelial databases from patients with defined eosinophilic T2 phenotypes and neutrophilic or pauci-granulocytic non-T2 phenotypes (117). Moreover, evaluation of datasets derived from patients with milder asthma could add relevant information, for example to confirm a potential role of RBP modulation by glucocorticoid therapy, so far identified in human airway epithelium *in vitro* (12).

There are several limitations also to the results obtained for the COPD database, the main one being the small number of subjects included. In support of our findings, however, this patient and control cohort did have statistical power to support the findings of the original study. We were able to validate our main finding of global downregulation of mRBPs in COPD/S in an independent small airway epithelial cell transcriptomic study (21), in particular for coexpressed mRBP genes whose changes were deemed by IPA analys to have a significant impact on several canonical pathways. It is nevertheless essential to evaluate other independent databases with larger number of COPD subjects and controls, possibly with detailed clinical phenotyping including alpha-1-antitrypsin deficiency status, length of smoking history or time from smoking cessation as well as history of exacerbation. However, a rate-limiting factor for this validation lies in the fact that few studies have focused on transcriptomic of small airway epithelium in large number of patients. While correlation network analysis is being conducted on the transcriptome of larger COPD case/control cohorts for which lung tissue samples are available (118), other systems-biology approaches using single-cell sequencing of small airway epithelial cells are concurrently uncovering relevant biological features using brushing samples isolated in a number of COPD and control subjects equal to, or smaller than that of the study we evaluated (119, 120).

Moreover, we searched epithelial GEO databases for a list of RBPs selected on the basis of canonical RBDs (20). It remains to be probed the profile of other RBPs that do not contain conventional RBDs (121), which have been increasingly identified in human RNA interactome studies. These RBPs also have RNA binding activity but hold several other functions, such enzymatic activity in metabolic pathways or in RNA modification; their role in human epithelial biology is largely unknown.

Overall, the COPD-related mRBP profile found in our study suggests post-transcriptional control of epithelial gene expression as substantial, yet understudied process possibly contributing to key pathogenic mechanisms in COPD. In-depth characterization of proteins dynamically interacting with mRNAs is necessary to understand how PTGR participates to the disease process – and whether it can be targeted therapeutically. Therefore, creating a map of RBP expression is a necessary first step to then analyze epithelial mRNA-bound proteome and potential changes in disease. Focused functional analysis and validating proteomic experiments will be needed to validate the coexpression of mRBPs and to address whether expression of mRBP targets, when known, in the COPD epithelial transcriptome database would show alterations consistent with – and dependent from - the documented changes of mRBPs expression.

## Data Availability Statement

The datasets presented in this study can be found in online repositories. The names of the repository/repositories and accession number(s) can be found in the article/Supplementary Material.

## Author Contributions

LR implemented all analyses, generated figures, and assisted in manuscript preparation. GG and DM bioinformatic and statistical analysis. JD, IS, and AN data extraction from GEO databases and analysis of specific datasets. MP generation of Tables and referencing. AV, GC, and VC selection of databases, data mining, and manuscript editing. CS study design and manuscript preparation. All authors contributed to the article and approved the submitted version.

## Conflict of Interest

The authors declare that the research was conducted in the absence of any commercial or financial relationships that could be construed as a potential conflict of interest.
