# Supplementary material for: Posttranscriptional Gene Regulatory Networks in Chronic Airway Inflammatory Diseases: In silico Mapping of RNA-Binding Protein Expression in Airway Epithelium
Source: Front Immunol. 2020 Oct 16;11:579889. doi: 10.3389/fimmu.2020.579889 (PMC7596416; doi:10.3389/fimmu.2020.579889)
Supplement: Supplementary file 1 [file Table_1.docx]

**Supplementary material**

**Table S1.** **Transcription factors identified by DNA binding motif search as putative regulators of mRBP genes downregulated in COPD/S (see Figure 6).**

| Gene Symbol | p-value |
| --- | --- |
| ZNF384 | 2.52E-11 |
| Arid5a | 1.10E-10 |
| MEF2C | 2.64E-09 |
| MEF2A | 9.36E-09 |
| Foxd3 | 1.38E-08 |
| MEF2D | 1.67E-07 |
| HOXD13 | 2.07E-07 |
| LIN54 | 1.10E-06 |
| HOXB13 | 4.67E-06 |
| HOXD11 | 9.82E-06 |
| ONECUT3 | 1.13E-05 |
| MEF2B | 1.47E-05 |
| PROP1 | 1.62E-05 |
| HOXA13 | 1.69E-05 |
| HOXA10 | 3.40E-05 |
| Arid3b | 0.000214762 |
| ONECUT2 | 0.000243658 |
| IRF7 | 0.000271221 |
| TBP | 0.000304726 |
| ONECUT1 | 0.000443501 |
| Lhx3 | 0.000497483 |
| Hoxd9 | 0.00053171 |
| HOXC13 | 0.000627864 |
| Foxj3 | 0.000751435 |
| HOXC10 | 0.00119655 |
| IRF1 | 0.00120053 |
| Arid3a | 0.00303598 |
| Dlx1 | 0.00355402 |
| HOXC12 | 0.00478733 |
| Foxq1 | 0.00546457 |
| Hoxa11 | 0.00566453 |
| OTX2 | 0.00815731 |
| E2F2 | 0.00848787 |
| OTX1 | 0.0126335 |
| CDX1 | 0.0135892 |
| FOXP2 | 0.013838 |
| FOXC2 | 0.0151089 |
| PAX3 | 0.0177663 |
| PAX7 | 0.0178403 |
| POU1F1 | 0.0199801 |
| mix-a | 0.0204305 |
| SOX15 | 0.0217705 |
| LMX1B | 0.0248127 |
| ALX3 | 0.0280069 |
| POU3F3 | 0.0313448 |
| Dmbx1 | 0.0367936 |
| Six3 | 0.0387961 |
| Phox2b | 0.0445245 |
| GRHL1 | 0.0446821 |
| MNX1 | 0.0450404 |

**Table S2.** **mRBPs gene clusters identified by correlated expression in COPD patients (COPD) versus NS and S in GSE5058 (see Figure 7).** **Probe ID = probes identify different gene portions on arrays.** FC = Fold change; FDR = False Discovery Rate. FDR ≤ 0.05 indicated in red. Color denotes an FC value ≤ -1.5 (green) and ≥ 1.5 (red).

| Cluster 1 | | |  |  |  |  |  |  |
| --- | --- | --- | --- | --- | --- | --- | --- | --- |
| Gene Symbol | **Probe ID** | **FC S vs NS** | | **FC COPD vs NS** | **FC COPD vs S** | **FDR S vs NS** | **FDR COPD vs NS** | **FDR COPD vs S** |
| HNRNPDL | 1554678_s_at | 1.06 | | **-1.67** | **-1.76** | 0.447328 | 0.0039532 | 0.00358 |
| RBM25 | 1557081_at | 1.37 | | **-4.16** | **-5.7** | 0.371572 | 0.0001691 | 0.0199102 |
| FXR1 | 201635_s_at | 1.13 | | **-3.06** | **-3.46** | 0.494398 | 0.0004195 | 0.0092905 |
| TPR | 201730_s_at | 1.39 | | **-2.9** | **-4.03** | 0.261883 | 0.002906 | 0.0071347 |
| SRSF1 | 201742_x_at | 1.02 | | **-3.46** | **-3.51** | 0.481943 | 0.0005547 | 0.012671 |
| HTATSF1 | 202601_s_at | 1.08 | | **-2.66** | **-2.89** | 0.494398 | 0.0025623 | 0.0102221 |
| TFAM | 203176_s_at | 1.31 | | **-2.02** | **-2.64** | 0.306115 | 0.0111959 | 0.0102221 |
| HNRNPR | 208765_s_at | -1.11 | | **-2.82** | **-2.55** | 0.263366 | 0.0048584 | 0.0092905 |
| LUC7L3 | 208835_s_at | 1.29 | | **-3.08** | **-3.97** | 0.470371 | 0.0019973 | 0.0150271 |
| CDC5L | 209055_s_at | -1.02 | | **-2.31** | **-2.27** | 0.40396 | 0.0038409 | 0.004293 |
| TNPO1 | 209226_s_at | 1.49 | | **-1.92** | **-2.87** | 0.146932 | 0.0212181 | 0.0190971 |
| NXT2 | 209629_s_at | 1.07 | | **-3.71** | **-3.97** | 0.470884 | 0.0001525 | 0.0133373 |
| SERBP1 | 209669_s_at | 1.08 | | **-1.86** | **-2.01** | 0.450844 | 0.0007262 | 0.0102221 |
| SRSF10 | 210178_x_at | 1.26 | | **-1.95** | **-2.47** | 0.451411 | 0.0024663 | 0.0142154 |
| RBM25 | 212028_at | 1.06 | | **-4.35** | **-4.63** | 0.486372 | 8.10E-06 | 0.0176927 |
| SRSF5 | 212266_s_at | 1.21 | | **-2.41** | **-2.92** | 0.355776 | 0.0011128 | 0.0161679 |
| THOC2 | 212994_at | 1.25 | | **-2.49** | **-3.12** | 0.287465 | 1.85E-05 | 0.0115967 |
| SRSF7 | 213649_at | 1.32 | | **-3.53** | **-4.65** | 0.337217 | 0.0007541 | 0.0180915 |
| API5 | 214959_s_at | -1.04 | | **-2.3** | **-2.22** | 0.451411 | 0.001405 | 0.0150271 |
| MTPAP | 218947_s_at | 1.05 | | **-2.25** | **-2.36** | 0.402346 | 8.81E-05 | 0.002677 |
| PNRC2 | 222406_s_at | 1.01 | | **-4.05** | **-4.1** | 0.448419 | 0.002339 | 0.0143254 |
| DHX36 | 223138_s_at | 1.08 | | **-2.58** | **-2.79** | 0.479051 | 0.0005561 | 0.0102221 |
| TNRC6B | 229036_at | 1.26 | | **-2.93** | **-3.7** | 0.253541 | 0.0007262 | 0.0107368 |
| MTPAP | 229676_at | 1.18 | | **-2.26** | **-2.68** | 0.492513 | 0.0130341 | 0.0233098 |
| CIRBP | 230142_s_at | 1.26 | | **-2.67** | **-3.35** | 0.439607 | 0.0060247 | 0.0308997 |
| DDX17 | 230180_at | 1.17 | | **-13.61** | **-15.92** | 0.352592 | 0.0001584 | 0.0254254 |
| GFM2 | 231917_at | 1.29 | | **-1.42** | **-1.83** | 0.303908 | 0.0084372 | 0.0150703 |
| GFM2 | 231918_s_at | 1.13 | | **-2.41** | **-2.73** | 0.393887 | 0.0003789 | 0.0121703 |
| GSPT1 | 234975_at | 1.06 | | **-1.68** | **-1.78** | 0.340616 | 0.0053674 | 0.018677 |
| SRSF3 | 235324_at | 1.15 | | **-2.23** | **-2.56** | 0.443723 | 0.0007541 | 0.030907 |
| WDR61 | 237208_at | 1.21 | | **-1.73** | **-2.09** | 0.326918 | 0.011176 | 0.0375516 |
| CCAR1 | 239014_at | -1.06 | | **-7.49** | **-7.07** | 0.435306 | 0.0019973 | 0.0226824 |
| SRSF4 | 239512_at | -1.05 | | **-3.11** | **-2.97** | 0.394863 | 0.0179043 | 0.0475534 |
| Cluster 2 | | |  |  |  |  |  |  |
| Gene Symbol | **Probe ID** | **FC S vs NS** | | **FC COPD vs NS** | **FC COPD vs S** | **FDR S vs NS** | **FDR COPD vs NS** | **FDR COPD vs S** |
| TIA1 | 1554890_a_at | **1.5** | | **-1.83** | **-2.75** | 0.213603 | 0.0185026 | 0.0286072 |
| LARP4 | 1555384_a_at | **1.44** | | **-2.52** | **-3.62** | 0.205514 | 0.0072113 | 0.0275965 |
| MBNL1 | 1555594_a_at | **2.05** | | **-1.87** | **-3.85** | 0.126396 | 0.0270442 | 0.018677 |
| LUC7L | 1557067_s_at | **1.27** | | **-1.89** | **-2.39** | 0.329217 | 0.0045248 | 0.018677 |
| YTHDF3 | 1564053_a_at | **1.27** | | **-2.49** | **-3.15** | 0.44673 | 0.0062783 | 0.0148378 |
| BCLAF1 | 201083_s_at | **1.6** | | **-1.58** | **-2.52** | 0.15161 | 0.0408363 | 0.0231177 |
| BCLAF1 | 201101_s_at | **1.77** | | **-4.13** | **-7.32** | 0.286402 | 0.0064546 | 0.0179086 |
| TIA1 | 201446_s_at | **1.49** | | **-3.84** | **-5.74** | 0.236813 | 0.0032023 | 0.0175399 |
| RANBP2 | 201711_x_at | **1.57** | | **-2.64** | **-4.15** | 0.186498 | 0.0008907 | 0.017659 |
| DZIP1 | 204556_s_at | **1.19** | | **-1.32** | **-1.57** | 0.213534 | 0.0643581 | 0.0297619 |
| MBNL2 | 205017_s_at | **1.38** | | **-2.57** | **-3.56** | 0.304615 | 0.0032023 | 0.030907 |
| MBNL2 | 205018_s_at | **1.34** | | **-3.01** | **-4.04** | 0.353503 | 0.0043656 | 0.0155975 |
| G3BP2 | 206383_s_at | **1.22** | | **-1.44** | **-1.75** | 0.230395 | 0.0243383 | 0.0323169 |
| SYNCRIP | 209024_s_at | **1.68** | | **-2.16** | **-3.64** | 0.106247 | 0.0005547 | 0.0092905 |
| RNGTT | 211849_s_at | **1.05** | | **-6.37** | **-6.68** | 0.413405 | 0.0051252 | 0.0221826 |
| DDX3X | 212514_x_at | **1.29** | | **-1.54** | **-1.98** | 0.189196 | 0.0474536 | 0.0345498 |
| BCLAF1 | 214499_s_at | **1.35** | | **-3.42** | **-4.63** | 0.316428 | 0.0008907 | 0.0150271 |
| PTBP3 | 214697_s_at | **-1** | | **-1.81** | **-1.8** | 0.399698 | 0.0102013 | 0.0221826 |
| GUF1 | 218884_s_at | **1.35** | | **-1.18** | **-1.59** | 0.250578 | 0.0900152 | 0.0271164 |
| ZRANB2 | 223016_x_at | **1.38** | | **-2.62** | **-3.63** | 0.306115 | 0.0009806 | 0.011906 |
| Cluster 3 | | |  |  |  |  |  |  |
| Gene Symbol | **Probe ID** | **FC S vs NS** | | **FC COPD vs NS** | **FC COPD vs S** | **FDR S vs NS** | **FDR COPD vs NS** | **FDR COPD vs S** |
| SCAF11 | 1570507_at | **-1.09** | | **-3.15** | **-2.88** | 0.467129 | 0.014375 | 0.0275015 |
| SF3B1 | 201071_x_at | **1.04** | | **-1.84** | **-1.91** | 0.499574 | 0.0046634 | 0.0121703 |
| TIA1 | 201449_at | **1** | | **-2.41** | **-2.42** | 0.491093 | 0.0149244 | 0.031216 |
| RANBP2 | 201712_s_at | **1.11** | | **-1.93** | **-2.15** | 0.36093 | 0.0137568 | 0.0114609 |
| RANBP2 | 201713_s_at | **1.17** | | **-1.6** | **-1.88** | 0.319399 | 0.0450322 | 0.0230986 |
| UPF2 | 203519_s_at | **-1.05** | | **-2.52** | **-2.4** | 0.404338 | 0.0053674 | 0.0188686 |
| WBP4 | 203599_s_at | **1.31** | | **-1.18** | **-1.54** | 0.265744 | 0.0628788 | 0.0232771 |
| SRSF10 | 204299_at | **-1.21** | | **-2.49** | **-2.07** | 0.376659 | 0.0242595 | 0.0468013 |
| DZIP1 | 204557_s_at | **-1.05** | | **-2.66** | **-2.54** | 0.354549 | 0.0156042 | 0.0190971 |
| CLK4 | 210346_s_at | **-1.1** | | **-2.62** | **-2.37** | 0.399698 | 0.0021015 | 0.0199102 |
| ZNF638 | 211257_x_at | **1.02** | | **-2.78** | **-2.82** | 0.395084 | 0.0021378 | 0.0150037 |
| PNN | 212036_s_at | **-1.17** | | **-4.93** | **-4.21** | 0.399274 | 0.0045727 | 0.0254254 |
| HNRNPDL | 212454_x_at | **1.07** | | **-2.19** | **-2.33** | 0.482769 | 0.0113383 | 0.0297528 |
| TNPO1 | 212635_at | **1.14** | | **-1.77** | **-2.01** | 0.486699 | 0.0304605 | 0.0202625 |
| SREK1 | 212721_at | **1.08** | | **-2.59** | **-2.81** | 0.487995 | 0.0150868 | 0.0319828 |
| FUBP1 | 212847_at | **1.06** | | **-1.85** | **-1.96** | 0.402346 | 0.0243383 | 0.0281825 |
| YTHDC2 | 213077_at | **-1.04** | | **-1.81** | **-1.74** | 0.467076 | 0.0451012 | 0.0332428 |
| HNRNPD | 213359_at | **1.32** | | **-2.69** | **-3.56** | 0.491093 | 0.0408363 | 0.0488046 |
| PPWD1 | 213483_at | **1.05** | | **-1.94** | **-2.04** | 0.41731 | 0.0150868 | 0.0224045 |
| FUBP1 | 214093_s_at | **1.3** | | **-3.21** | **-4.17** | 0.407856 | 0.008399 | 0.0141766 |
| SRSF7 | 214141_x_at | **1.12** | | **-2.07** | **-2.32** | 0.482769 | 0.0003094 | 0.0133373 |
| CLK1 | 214683_s_at | **-1.07** | | **-3.78** | **-3.55** | 0.342525 | 0.0040844 | 0.025705 |
| SLTM | 217828_at | **1.06** | | **-1.64** | **-1.75** | 0.492836 | 0.0274489 | 0.0397468 |
| CAPRIN2 | 218456_at | **-1.24** | | **-2.21** | **-1.78** | 0.304049 | 0.0021378 | 0.0353238 |
| LUC7L3 | 220044_x_at | **1.01** | | **-2.36** | **-2.38** | 0.47383 | 0.0164155 | 0.0181931 |
| SFPQ | 221768_at | **1.01** | | **-5.11** | **-5.18** | 0.39481 | 0.0067192 | 0.0275965 |
| ANGEL2 | 221826_at | **-1.01** | | **-1.7** | **-1.68** | 0.496762 | 0.0027824 | 0.0221826 |
| HNRNPA1 | 221919_at | **-1.26** | | **-2.83** | **-2.26** | 0.234404 | 0.0053674 | 0.0388807 |
| DHX36 | 223140_s_at | **1** | | **-1.72** | **-1.72** | 0.479051 | 0.020492 | 0.0180915 |
| ZRANB2 | 223716_s_at | **1.22** | | **-2.29** | **-2.78** | 0.346255 | 0.0360208 | 0.0188656 |
| TNRC6A | 224705_s_at | **-1.02** | | **-3.19** | **-3.14** | 0.381743 | 0.0036641 | 0.025705 |
| NUFIP2 | 224938_at | **1.01** | | **-1.89** | **-1.91** | 0.423526 | 0.0300288 | 0.0162441 |
| HNRNPA2B1 | 225107_at | **1.11** | | **-4.43** | **-4.91** | 0.487983 | 0.0114244 | 0.0251596 |
| RC3H1 | 225893_at | **1.14** | | **-1.94** | **-2.21** | 0.491755 | 0.0132038 | 0.0308894 |
| HELZ | 225910_at | **-1.22** | | **-1.94** | **-1.59** | 0.44806 | 0.0113383 | 0.0311165 |
| MSI2 | 226134_s_at | **1.04** | | **-1.66** | **-1.72** | 0.492836 | 0.0209936 | 0.0150271 |
| SRSF1 | 226419_s_at | **1.05** | | **-2.16** | **-2.26** | 0.411642 | 0.0090966 | 0.0428733 |
| PPIL4 | 226472_at | **-1.11** | | **-2.25** | **-2.03** | 0.496762 | 0.0113383 | 0.0115967 |
| TPR | 228709_at | **-1.03** | | **-2.02** | **-1.95** | 0.484297 | 0.0090674 | 0.0223358 |
| RBM26 | 229433_at | **1.23** | | **-1.84** | **-2.26** | 0.403047 | 0.0164155 | 0.0278978 |
| SREK1 | 235611_at | **1.07** | | **-1.5** | **-1.61** | 0.346146 | 0.1361973 | 0.0467 |
| ZNF326 | 236196_at | **1.17** | | **-1.57** | **-1.83** | 0.371045 | 0.0396157 | 0.0332428 |
| DZIP1L | 239785_at | **-1.1** | | **-2.12** | **-1.92** | 0.367387 | 0.0140181 | 0.0453026 |
| Cluster 4 | | |  |  |  |  |  |  |
| Gene Symbol | **Probe ID** | **FC S vs NS** | | **FC COPD vs NS** | **FC COPD vs S** | **FDR S vs NS** | **FDR COPD vs NS** | **FDR COPD vs S** |
| PNN | 1567214_a_at | **-1.12** | | **-3.15** | **-2.82** | 0.319227 | 0.0124289 | 0.0234638 |
| DEK | 200934_at | **-1.1** | | **-1.89** | **-1.72** | 0.426825 | 0.0377842 | 0.0350833 |
| IPO7 | 200995_at | **1.05** | | **-1.45** | **-1.52** | 0.459827 | 0.0926571 | 0.0429633 |
| SF3B1 | 201071_x_at | **1.04** | | **-1.84** | **-1.91** | 0.499574 | 0.0046634 | 0.0121703 |
| BCLAF1 | 201084_s_at | **-1.01** | | **-2.05** | **-2.03** | 0.418611 | 0.0134253 | 0.01625 |
| SRSF7 | 201129_at | **1.16** | | **-1.34** | **-1.54** | 0.413405 | 0.0041782 | 0.0296859 |
| TIA1 | 201447_at | **1.35** | | **-2.28** | **-3.08** | 0.357185 | 0.0102013 | 0.0268711 |
| TIA1 | 201449_at | **1** | | **-2.41** | **-2.42** | 0.491093 | 0.0149244 | 0.031216 |
| TIA1 | 201450_s_at | **1.38** | | **-2.19** | **-3.02** | 0.352592 | 0.0184955 | 0.025705 |
| PUM2 | 201493_s_at | **-1.04** | | **-1.75** | **-1.68** | 0.311061 | 0.0021999 | 0.0204315 |
| RANBP2 | 201712_s_at | **1.11** | | **-1.93** | **-2.15** | 0.36093 | 0.0137568 | 0.0114609 |
| RANBP2 | 201713_s_at | **1.17** | | **-1.6** | **-1.88** | 0.319399 | 0.0450322 | 0.0230986 |
| SETX | 201964_at | **1.1** | | **-1.68** | **-1.85** | 0.253103 | 0.0041782 | 0.0092905 |
| PCF11 | 203378_at | **1.09** | | **-2.19** | **-2.4** | 0.494398 | 0.0032023 | 0.0092905 |
| UPF2 | 203519_s_at | **-1.05** | | **-2.52** | **-2.4** | 0.404338 | 0.0053674 | 0.0188686 |
| WBP4 | 203599_s_at | **1.31** | | **-1.18** | **-1.54** | 0.265744 | 0.0628788 | 0.0232771 |
| FMR1 | 203689_s_at | **-1** | | **-1.52** | **-1.51** | 0.481123 | 0.0194288 | 0.0288196 |
| SRSF10 | 204299_at | **-1.21** | | **-2.49** | **-2.07** | 0.376659 | 0.0242595 | 0.0468013 |
| DZIP1 | 204557_s_at | **-1.05** | | **-2.66** | **-2.54** | 0.354549 | 0.0156042 | 0.0190971 |
| SRSF10 | 206095_s_at | **-1.01** | | **-1.76** | **-1.74** | 0.286119 | 0.0177866 | 0.0190971 |
| UPF3A | 206958_s_at | **-1.18** | | **-2.44** | **-2.06** | 0.16421 | 0.0039057 | 0.030907 |
| XPO1 | 208775_at | **1.18** | | **-1.33** | **-1.57** | 0.306115 | 0.0893477 | 0.0473632 |
| SRSF1 | 208863_s_at | **1.04** | | **-1.47** | **-1.52** | 0.269899 | 0.0214501 | 0.011906 |
| HNRNPH3 | 208990_s_at | **-1.01** | | **-1.56** | **-1.54** | 0.404338 | 0.0179043 | 0.0226824 |
| HNRNPDL | 209068_at | **1.05** | | **-1.6** | **-1.69** | 0.394863 | 0.0185026 | 0.0254254 |
| CLK4 | 210346_s_at | **-1.1** | | **-2.62** | **-2.37** | 0.399698 | 0.0021015 | 0.0199102 |
| ZNF638 | 211257_x_at | **1.02** | | **-2.78** | **-2.82** | 0.395084 | 0.0021378 | 0.0150037 |
| HNRNPA3 | 211929_at | **-1.13** | | **-2.1** | **-1.85** | 0.219654 | 0.0135295 | 0.0443549 |
| RBM25 | 212031_at | **1.16** | | **-1.78** | **-2.06** | 0.382279 | 0.0135295 | 0.018677 |
| PNN | 212036_s_at | **-1.17** | | **-4.93** | **-4.21** | 0.399274 | 0.0045727 | 0.0254254 |
| HNRNPDL | 212454_x_at | **1.07** | | **-2.19** | **-2.33** | 0.482769 | 0.0113383 | 0.0297528 |
| TNPO1 | 212635_at | **1.14** | | **-1.77** | **-2.01** | 0.486699 | 0.0304605 | 0.0202625 |
| LARP4 | 212714_at | **1.13** | | **-2.29** | **-2.59** | 0.499255 | 0.0063002 | 0.0190971 |
| SREK1 | 212721_at | **1.08** | | **-2.59** | **-2.81** | 0.487995 | 0.0150868 | 0.0319828 |
| FUBP1 | 212847_at | **1.06** | | **-1.85** | **-1.96** | 0.402346 | 0.0243383 | 0.0281825 |
| DCP2 | 212919_at | **1.11** | | **-1.71** | **-1.9** | 0.482322 | 0.0135295 | 0.0150271 |
| ZC3H14 | 213063_at | **-1.04** | | **-1.77** | **-1.7** | 0.354478 | 0.0209311 | 0.026575 |
| YTHDC2 | 213077_at | **-1.04** | | **-1.81** | **-1.74** | 0.467076 | 0.0451012 | 0.0332428 |
| DZIP3 | 213186_at | **1.3** | | **-1.5** | **-1.95** | 0.093215 | 0.1499942 | 0.0162104 |
| HNRNPD | 213359_at | **1.32** | | **-2.69** | **-3.56** | 0.491093 | 0.0408363 | 0.0488046 |
| PPWD1 | 213483_at | **1.05** | | **-1.94** | **-2.04** | 0.41731 | 0.0150868 | 0.0224045 |
| SFPQ | 214016_s_at | **-1.06** | | **-2.77** | **-2.61** | 0.230123 | 0.0068489 | 0.0332428 |
| FUBP1 | 214093_s_at | **1.3** | | **-3.21** | **-4.17** | 0.407856 | 0.008399 | 0.0141766 |
| CLK1 | 214683_s_at | **-1.07** | | **-3.78** | **-3.55** | 0.342525 | 0.0040844 | 0.025705 |
| FMR1 | 215245_x_at | **1.03** | | **-1.57** | **-1.61** | 0.470371 | 0.0161524 | 0.0346335 |
| SLTM | 217828_at | **1.06** | | **-1.64** | **-1.75** | 0.492836 | 0.0274489 | 0.0397468 |
| CAPRIN2 | 218456_at | **-1.24** | | **-2.21** | **-1.78** | 0.304049 | 0.0021378 | 0.0353238 |
| ZCCHC8 | 218478_s_at | **-1** | | **-1.5** | **-1.5** | 0.271713 | 0.0035644 | 0.026575 |
| LUC7L3 | 220044_x_at | **1.01** | | **-2.36** | **-2.38** | 0.47383 | 0.0164155 | 0.0181931 |
| TARDBP | 221264_s_at | **1.18** | | **-1.74** | **-2.06** | 0.417059 | 0.0265162 | 0.0254254 |
| SFPQ | 221768_at | **1.01** | | **-5.11** | **-5.18** | 0.39481 | 0.0067192 | 0.0275965 |
| HNRNPA1 | 221919_at | **-1.26** | | **-2.83** | **-2.26** | 0.234404 | 0.0053674 | 0.0388807 |
| HNRNPA1 | 222040_at | **-1.22** | | **-2.55** | **-2.09** | 0.247381 | 0.0169117 | 0.0499241 |
| MYEF2 | 222772_at | **-1.12** | | **-1.92** | **-1.72** | 0.429223 | 0.03467 | 0.0337946 |
| DHX36 | 223140_s_at | **1** | | **-1.72** | **-1.72** | 0.479051 | 0.020492 | 0.0180915 |
| ZRANB2 | 223716_s_at | **1.22** | | **-2.29** | **-2.78** | 0.346255 | 0.0360208 | 0.0188656 |
| UHMK1 | 224691_at | **1.09** | | **-1.38** | **-1.51** | 0.451411 | 0.0653394 | 0.0328503 |
| TNRC6A | 224705_s_at | **-1.02** | | **-3.19** | **-3.14** | 0.381743 | 0.0036641 | 0.025705 |
| NUDT21 | 224830_at | **-1.01** | | **-1.75** | **-1.72** | 0.496762 | 0.0291724 | 0.0301838 |
| NUFIP2 | 224938_at | **1.01** | | **-1.89** | **-1.91** | 0.423526 | 0.0300288 | 0.0162441 |
| HNRNPA2B1 | 225107_at | **1.11** | | **-4.43** | **-4.91** | 0.487983 | 0.0114244 | 0.0251596 |
| SCAF11 | 225336_at | **-1.01** | | **-1.89** | **-1.87** | 0.43975 | 0.0265162 | 0.0275015 |
| PAPD4 | 225761_at | **1.02** | | **-1.52** | **-1.55** | 0.375965 | 0.0144164 | 0.0214437 |
| RC3H1 | 225893_at | **1.14** | | **-1.94** | **-2.21** | 0.491755 | 0.0132038 | 0.0308894 |
| HELZ | 225910_at | **-1.22** | | **-1.94** | **-1.59** | 0.44806 | 0.0113383 | 0.0311165 |
| HNRNPA2B1 | 225932_s_at | **-1.26** | | **-3.85** | **-3.05** | 0.168412 | 0.0091458 | 0.0274007 |
| MSI2 | 226134_s_at | **1.04** | | **-1.66** | **-1.72** | 0.492836 | 0.0209936 | 0.0150271 |
| RBM26 | 226316_at | **1.22** | | **-2.32** | **-2.81** | 0.379267 | 0.0144164 | 0.0323119 |
| SRSF1 | 226419_s_at | **1.05** | | **-2.16** | **-2.26** | 0.411642 | 0.0090966 | 0.0428733 |
| PPIL4 | 226472_at | **-1.11** | | **-2.25** | **-2.03** | 0.496762 | 0.0113383 | 0.0115967 |
| CWC22 | 226588_at | **-1.13** | | **-2.02** | **-1.78** | 0.40396 | 0.0147103 | 0.0127743 |
| PABPC1L | 226670_s_at | **1.02** | | **-2.67** | **-2.73** | 0.288091 | 0.0286504 | 0.0333488 |
| RANBP2 | 226922_at | **1.11** | | **-1.55** | **-1.71** | 0.375965 | 0.0483165 | 0.0286315 |
| CPEB2 | 226939_at | **-1.01** | | **-1.58** | **-1.56** | 0.382279 | 0.0234358 | 0.0204315 |
| TPR | 228709_at | **-1.03** | | **-2.02** | **-1.95** | 0.484297 | 0.0090674 | 0.0223358 |
| CLK4 | 228751_at | **-1.3** | | **-2.32** | **-1.79** | 0.280005 | 0.0096155 | 0.0405681 |
| RBM26 | 229433_at | **1.23** | | **-1.84** | **-2.26** | 0.403047 | 0.0164155 | 0.0278978 |
| MYEF2 | 229464_at | **1.15** | | **-1.85** | **-2.13** | 0.417059 | 0.0404116 | 0.0389671 |
| RAVER2 | 231851_at | **-1.05** | | **-1.86** | **-1.77** | 0.352592 | 0.020866 | 0.0306465 |
| MYEF2 | 232676_x_at | **1.04** | | **-2.25** | **-2.34** | 0.394863 | 0.0084147 | 0.0142029 |
| SCAF11 | 235579_at | **-1.04** | | **-1.6** | **-1.54** | 0.390845 | 0.0172984 | 0.0390701 |
| SREK1 | 235611_at | **1.07** | | **-1.5** | **-1.61** | 0.346146 | 0.1361973 | 0.0467 |
| ZNF326 | 236196_at | **1.17** | | **-1.57** | **-1.83** | 0.371045 | 0.0396157 | 0.0332428 |
| RBM20 | 238763_at | **-1.3** | | **-3.89** | **-3** | 0.230395 | 0.0078456 | 0.0254254 |
| SREK1 | 238781_at | **1.16** | | **-1.61** | **-1.86** | 0.394863 | 0.0410982 | 0.0355599 |
| Cluster 5 | | |  |  |  |  |  |  |
| Gene Symbol | **Probe ID** | **FC S vs NS** | | **FC COPD vs NS** | **FC COPD vs S** | **FDR S vs NS** | **FDR COPD vs NS** | **FDR COPD vs S** |
| PNN | 1567214_a_at | **-1.12** | | **-3.15** | **-2.82** | 0.319227 | 0.0124289 | 0.0234638 |
| DEK | 200934_at | **-1.1** | | **-1.89** | **-1.72** | 0.426825 | 0.0377842 | 0.0350833 |
| IPO7 | 200995_at | **1.05** | | **-1.45** | **-1.52** | 0.459827 | 0.0926571 | 0.0429633 |
| BCLAF1 | 201084_s_at | **-1.01** | | **-2.05** | **-2.03** | 0.418611 | 0.0134253 | 0.01625 |
| SRSF7 | 201129_at | **1.16** | | **-1.34** | **-1.54** | 0.413405 | 0.0041782 | 0.0296859 |
| TIA1 | 201447_at | **1.35** | | **-2.28** | **-3.08** | 0.357185 | 0.0102013 | 0.0268711 |
| TIA1 | 201450_s_at | **1.38** | | **-2.19** | **-3.02** | 0.352592 | 0.0184955 | 0.025705 |
| PUM2 | 201493_s_at | **-1.04** | | **-1.75** | **-1.68** | 0.311061 | 0.0021999 | 0.0204315 |
| SETX | 201964_at | **1.1** | | **-1.68** | **-1.85** | 0.253103 | 0.0041782 | 0.0092905 |
| PCF11 | 203378_at | **1.09** | | **-2.19** | **-2.4** | 0.494398 | 0.0032023 | 0.0092905 |
| FMR1 | 203689_s_at | **-1** | | **-1.52** | **-1.51** | 0.481123 | 0.0194288 | 0.0288196 |
| SRSF10 | 206095_s_at | **-1.01** | | **-1.76** | **-1.74** | 0.286119 | 0.0177866 | 0.0190971 |
| UPF3A | 206958_s_at | **-1.18** | | **-2.44** | **-2.06** | 0.16421 | 0.0039057 | 0.030907 |
| XPO1 | 208775_at | **1.18** | | **-1.33** | **-1.57** | 0.306115 | 0.0893477 | 0.0473632 |
| HNRNPH3 | 208990_s_at | **-1.01** | | **-1.56** | **-1.54** | 0.404338 | 0.0179043 | 0.0226824 |
| HNRNPDL | 209068_at | **1.05** | | **-1.6** | **-1.69** | 0.394863 | 0.0185026 | 0.0254254 |
| HNRNPA3 | 211929_at | **-1.13** | | **-2.1** | **-1.85** | 0.219654 | 0.0135295 | 0.0443549 |
| RBM25 | 212031_at | **1.16** | | **-1.78** | **-2.06** | 0.382279 | 0.0135295 | 0.018677 |
| LARP4 | 212714_at | **1.13** | | **-2.29** | **-2.59** | 0.499255 | 0.0063002 | 0.0190971 |
| DCP2 | 212919_at | **1.11** | | **-1.71** | **-1.9** | 0.482322 | 0.0135295 | 0.0150271 |
| ZC3H14 | 213063_at | **-1.04** | | **-1.77** | **-1.7** | 0.354478 | 0.0209311 | 0.026575 |
| DZIP3 | 213186_at | **1.3** | | **-1.5** | **-1.95** | 0.093215 | 0.1499942 | 0.0162104 |
| SFPQ | 214016_s_at | **-1.06** | | **-2.77** | **-2.61** | 0.230123 | 0.0068489 | 0.0332428 |
| FMR1 | 215245_x_at | **1.03** | | **-1.57** | **-1.61** | 0.470371 | 0.0161524 | 0.0346335 |
| ZCCHC8 | 218478_s_at | **-1** | | **-1.5** | **-1.5** | 0.271713 | 0.0035644 | 0.026575 |
| TARDBP | 221264_s_at | **1.18** | | **-1.74** | **-2.06** | 0.417059 | 0.0265162 | 0.0254254 |
| HNRNPA1 | 222040_at | **-1.22** | | **-2.55** | **-2.09** | 0.247381 | 0.0169117 | 0.0499241 |
| MYEF2 | 222772_at | **-1.12** | | **-1.92** | **-1.72** | 0.429223 | 0.03467 | 0.0337946 |
| PAIP2 | 222984_at | **1.2** | | **-1.32** | **-1.58** | 0.24807 | 0.1362808 | 0.025838 |
| UHMK1 | 224691_at | **1.09** | | **-1.38** | **-1.51** | 0.451411 | 0.0653394 | 0.0328503 |
| NUDT21 | 224830_at | **-1.01** | | **-1.75** | **-1.72** | 0.496762 | 0.0291724 | 0.0301838 |
| SCAF11 | 225336_at | **-1.01** | | **-1.89** | **-1.87** | 0.43975 | 0.0265162 | 0.0275015 |
| PAPD4 | 225761_at | **1.02** | | **-1.52** | **-1.55** | 0.375965 | 0.0144164 | 0.0214437 |
| HNRNPA2B1 | 225932_s_at | **-1.26** | | **-3.85** | **-3.05** | 0.168412 | 0.0091458 | 0.0274007 |
| RBM26 | 226316_at | **1.22** | | **-2.32** | **-2.81** | 0.379267 | 0.0144164 | 0.0323119 |
| CWC22 | 226588_at | **-1.13** | | **-2.02** | **-1.78** | 0.40396 | 0.0147103 | 0.0127743 |
| PABPC1L | 226670_s_at | **1.02** | | **-2.67** | **-2.73** | 0.288091 | 0.0286504 | 0.0333488 |
| RANBP2 | 226922_at | **1.11** | | **-1.55** | **-1.71** | 0.375965 | 0.0483165 | 0.0286315 |
| CPEB2 | 226939_at | **-1.01** | | **-1.58** | **-1.56** | 0.382279 | 0.0234358 | 0.0204315 |
| CLK4 | 228751_at | **-1.3** | | **-2.32** | **-1.79** | 0.280005 | 0.0096155 | 0.0405681 |
| MYEF2 | 229464_at | **1.15** | | **-1.85** | **-2.13** | 0.417059 | 0.0404116 | 0.0389671 |
| RAVER2 | 231851_at | **-1.05** | | **-1.86** | **-1.77** | 0.352592 | 0.020866 | 0.0306465 |
| MYEF2 | 232676_x_at | **1.04** | | **-2.25** | **-2.34** | 0.394863 | 0.0084147 | 0.0142029 |
| SCAF11 | 235579_at | **-1.04** | | **-1.6** | **-1.54** | 0.390845 | 0.0172984 | 0.0390701 |
| RBM20 | 238763_at | **-1.3** | | **-3.89** | **-3** | 0.230395 | 0.0078456 | 0.0254254 |
| SREK1 | 238781_at | **1.16** | | **-1.61** | **-1.86** | 0.394863 | 0.0410982 | 0.0355599 |

**Table S3.** Original clinical, spirometric and peripheral blood parameters of the full study cohorts providing airway epithelial cells by bronchial brushings for trascriptomic analysis reported in ([1](#_ENREF_1)), from which HC and SA(listed as Severe) datasets were extracted from GEO GSE63142 for analysis of mRBP expression in this study. Reprinted from ([1](#_ENREF_1)), with permission.

**
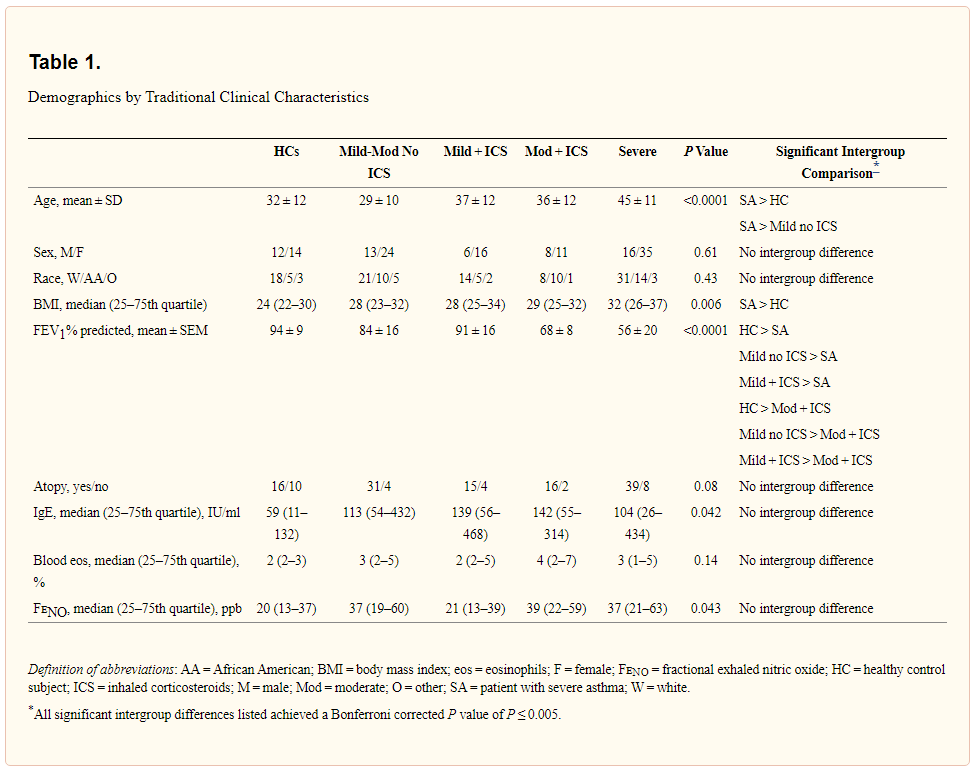
**

**Table S4.** List of mRBPs DEG genes in severe asthma (SA) versus healthy controls (HCs) (GSE63142 database). FC = Fold change; FDR = False Discovery Rate. The red text denotes an FDR ≤ 0.05.

| Gene Symbol | Probe ID | FC | FDR |
| --- | --- | --- | --- |
| DHX35 | A_23_P5945 | 1.09 | 0.000074 |
| IGF2BP3 | A_23_P19987 | 1.08 | 0.002246 |
| DQX1 | A_23_P56659 | 1.07 | 0.000004 |
| CELF2 | A_23_P202071 | 1.05 | 0.002258 |
| DQX1 | A_24_P68814 | 1.04 | 0.000019 |
| CCRN4L | A_24_P213794 | 1.03 | 0.00546 |
| HDLBP | A_23_P142804 | 1.03 | 0.020226 |
| CELF2 | A_23_P115645 | 1.03 | 0.014455 |
| NOL3 | A_23_P206371 | 1.03 | 0.000477 |
| APOBEC3F | A_23_P369966 | 1.03 | 0.014455 |
| THOC5 | A_23_P109470 | 1.03 | 0.025766 |
| CPSF4 | A_23_P42997 | 1.02 | 0.002193 |
| SMG9 | A_23_P56127 | 1.02 | 0.00228 |
| EIF4E2 | A_23_P165722 | 1.02 | 0.002246 |
| SPATS2 | A_23_P99303 | 1.02 | 0.004251 |
| NANOS1 | A_24_P383609 | 1.02 | 0.042071 |
| WBP4 | A_23_P140035 | 1.02 | 0.045296 |
| EIF2AK3 | A_23_P135857 | 1.02 | 0.02908 |
| EIF4E3 | A_23_P406425 | 1.02 | 0.009381 |
| AKAP1 | A_32_P132438 | 1.02 | 0.020444 |
| FAM98A | A_23_P313728 | 1.02 | 0.035518 |
| CRNKL1 | A_24_P40229 | 1.02 | 0.008604 |
| IPO11 | A_23_P30264 | 1.02 | 0.034467 |
| GAPDH | A_23_P13899 | 1.01 | 0.017944 |
| RBM45 | A_23_P142994 | 1.01 | 0.025766 |
| MSI2 | A_23_P369479 | 1.01 | 0.041039 |
| RBM18 | A_23_P20777 | 1.01 | 0.039068 |
| PELO | A_23_P58763 | 1.01 | 0.042071 |
| HNRNPM | A_32_P99549 | 1.01 | 0.023455 |
| NCBP1 | A_23_P257104 | 1.01 | 0.027085 |
| POLR2G | A_23_P36147 | 1.01 | 0.025766 |
| MAGOHB | A_24_P828949 | 1.01 | 0.031663 |
| ILF3 | A_23_P218456 | -1.01 | 0.008286 |
| PCBP2 | A_23_P204052 | -1.01 | 0.038925 |
| YTHDC1 | A_24_P208961 | -1.01 | 0.038913 |
| GRSF1 | A_23_P212937 | -1.01 | 0.020226 |
| CHTOP | A_23_P201726 | -1.01 | 0.022568 |
| U2AF1 | A_24_P917934 | -1.01 | 0.031663 |
| TARDBP | A_23_P403955 | -1.02 | 0.039451 |
| PAIP1 | A_23_P207811 | -1.02 | 0.039068 |
| CAPRIN2 | A_24_P400675 | -1.02 | 0.017944 |
| TNRC6A | A_23_P349310 | -1.02 | 0.023654 |
| HNRNPU | A_32_P51894 | -1.02 | 0.009381 |
| RBM23 | A_24_P207091 | -1.02 | 0.017944 |
| KHDRBS1 | A_23_P149012 | -1.02 | 0.034626 |
| SPEN | A_23_P12053 | -1.02 | 0.041039 |
| CIRBP | A_23_P142322 | -1.02 | 0.008286 |
| ZFP36L1 | A_23_P99540 | -1.02 | 0.015611 |
| LUC7L2 | A_23_P59791 | -1.02 | 0.031663 |
| RBM23 | A_23_P88249 | -1.02 | 0.020444 |
| LUC7L2 | A_23_P59787 | -1.02 | 0.036593 |
| TAF15 | A_24_P928068 | -1.02 | 0.020226 |
| MECP2 | A_24_P237486 | -1.02 | 0.020248 |
| RBMS1 | A_23_P36496 | -1.02 | 0.025766 |
| HNRNPH3 | A_23_P115824 | -1.03 | 0.020226 |
| EIF1B | A_23_P6891 | -1.03 | 0.020226 |
| ADARB1 | A_23_P211207 | -1.03 | 0.002246 |
| MYEF2 | A_23_P77079 | -1.03 | 0.008604 |
| CAPRIN2 | A_24_P56281 | -1.03 | 0.025766 |
| DDX17 | A_24_P925611 | -1.03 | 0.00963 |
| ELAVL4 | A_24_P188447 | -1.03 | 0.019424 |
| SRRM4 | A_23_P391479 | -1.03 | 0.020519 |
| CAPRIN2 | A_23_P87532 | -1.03 | 0.016679 |
| SF1 | A_24_P235783 | -1.03 | 0.009325 |
| SRSF7 | A_24_P222911 | -1.03 | 0.002416 |
| PABPC1L | A_24_P92183 | -1.04 | 0.019424 |
| BICC1 | A_32_P119348 | -1.05 | 0.020226 |
| FTO | A_23_P113184 | -1.05 | 0.002416 |

**References**

1. Modena BD, Tedrow JR, Milosevic J, Bleecker ER, Meyers DA, Wu W, et al. Gene expression in relation to exhaled nitric oxide identifies novel asthma phenotypes with unique biomolecular pathways. American Journal of Respiratory and Critical Care Medicine. 2014;190(12):1363-72.
